# Effectiveness of Mobile Health for Improving Medication Adherence in Patients With Cancer: Systematic Review and Meta-Analysis of Randomized Controlled Trials

**DOI:** 10.2196/85949

**Published:** 2026-03-30

**Authors:** Wei Chao Liu, Li Ping Wang, Qian Yu Zhang, Jing Han Yang

**Affiliations:** 1 School of Public Health and Nursing, Hangzhou Normal University, No. 2318 Yuhangtang Road, Hangzhou Normal University Cangqian Campus, Hangzhou, 311121, China, 86 0571 2886 1971; 2 Engineering Research Center of Mobile Health Management System, Ministry of Education, Hangzhou, China

**Keywords:** mobile health, medication adherence, cancer, randomized controlled trial, systematic review, meta-analysis

## Abstract

**Background:**

Medication adherence among patients with cancer is generally low. Mobile health (mHealth) has gradually been applied to improve this situation, but systematic evidence of its effectiveness remains lacking.

**Objective:**

We aimed to evaluate the effect of mHealth on improving medication adherence among patients with cancer.

**Methods:**

This systematic review included randomized controlled trials (RCTs) evaluating the impact of mHealth on medication adherence among patients with cancer. Systematic searches were conducted in PubMed, Web of Science, CINAHL, Cochrane Library, Embase, Sinomed, CNKI, Cqvip, and ClinicalTrials.gov from inception to December 31, 2025. Two researchers independently performed literature screening, data extraction, and risk of bias assessment. Effects were pooled using a random-effects model (Hartung-Knapp-Sidik-Jonkman), and standardized mean differences (SMDs) and odds ratios (ORs) with 95% CIs have been reported. Evidence quality was assessed using the GRADE (Grading of Recommendations Assessment, Development, and Evaluation) framework.

**Results:**

A total of 17 RCTs (1309 participants) from 8 countries published between 2016 and 2025 were included. mHealth interventions included mobile apps, websites, and text messaging services. The meta-analysis revealed that compared with controls, mHealth interventions significantly improved medication adherence rates (OR 3.47, 95% CI 1.92-6.26; *P*=.002), medication adherence scores (SMD 1.01, 95% CI 0.51-1.52; *P*=.001), self-efficacy (SMD 0.90, 95% CI 0.29-1.51; *P*=.01), and service satisfaction while reducing symptom burden (SMD −0.38, 95% CI −0.61 to −0.14; *P*=.008). However, mHealth had no significant effect on health literacy (SMD 0.51, 95% CI –1.50 to 2.52; *P*=.29). Subgroup analysis revealed that interventions lasting <3 months outperformed those lasting ≥3 months in improving adherence scores (SMD 1.37, 95% CI 0.78-1.96 vs SMD 0.49, 95% CI −0.39 to 1.37; *χ*²_1_=5.98; *P*=.01). Regarding intervention format, text messaging services demonstrated superior efficacy compared with mobile apps and websites (SMD 1.53, 95% CI −5.49 to 8.55 vs SMD 1.01, 95% CI 0.42-1.61 and SMD 0.11, 95% CI −0.34 to 0.56, respectively; *χ*²_2_=10.28; *P*=.006). Across cancer types, mHealth most significantly improved adherence scores in patients with breast cancer (SMD 1.29, 95% CI −5.25 to 7.83), outperforming the findings in patients with leukemia and other cancer types (SMD 0.28, 95% CI −0.87 to 1.42 and SMD 1.09, 95% CI 0.10-2.08, respectively; *χ*²_2_=8.86; *P*=.01).

**Conclusions:**

Our findings confirm that mHealth plays a positive role in improving medication adherence, enhancing patient self-efficacy, increasing patient satisfaction with services, and alleviating symptom burden. However, these findings should be interpreted with caution owing to substantial heterogeneity, a moderate risk of bias, and a low certainty of evidence. Future research should enhance methodological quality by conducting multicenter, large-sample, high-quality RCTs and should explore the long-term effects and cost-effectiveness of mHealth across diverse health care settings and patient populations to clarify its role and value within comprehensive cancer care management systems.

## Introduction

With the continuous rise in global cancer incidence, cancer has become one of the major public health threats to human health. In recent years, the rapid advancement of targeted therapy, immunotherapy, and endocrine therapy has led to the increasingly widespread clinical application of oral anticancer drugs [1]. Compared with intravenous treatment, oral therapy offers advantages, such as convenient administration and lower cost, making it a first-line treatment option in comprehensive cancer management [2]. Statistics indicate that approximately 60% of patients with cancer require 12‐24 weeks of oral endocrine therapy, targeted therapy, immunotherapy, or chemotherapy [3].

Medication adherence refers to whether patients take their drugs as prescribed by health care professionals and whether they consistently continue the prescribed medications [4]. For oral therapies, treatment efficacy is largely dependent on patient adherence [5]. Unlike intravenous therapy, oral treatment relies primarily on patients and caregivers administering medications independently at home, lacking real-time supervision by health care providers. Compounded by factors, such as prolonged treatment duration, significant drug toxicity and side effects, heavy financial burden, and increased psychological stress, patients with cancer are prone to behaviors such as missed doses, unauthorized dose adjustment, and premature discontinuation [6,7]. Studies indicate that medication adherence among patients with cancer is below 50%, reflecting an overall low level [8].

Poor medication adherence not only reduces the effectiveness of oral therapy but may also cause a series of adverse effects for patients. First, regarding symptom control, patients who fail to take medication regularly or who arbitrarily adjust dosages are prone to fluctuations in blood drug concentrations. This makes it difficult to achieve sustained suppression of tumor growth, thereby diminishing treatment efficacy and exacerbating the burden of symptoms, such as pain, fatigue, and nausea, severely impacting the quality of life of patients [9]. Second, regarding treatment safety, poor medication adherence may increase the risk of adverse events. Failure to follow prescribed regimens not only disrupts established treatment schedules but may also result in insufficient accumulation of effective drug doses, complicating the pattern of adverse reaction occurrence. Clinicians struggle to accurately distinguish drug-induced adverse reactions from natural disease progression, impairing clinical judgment and treatment decisions. This delays optimal intervention timing and increases the likelihood of patients requiring emergency care or unplanned hospitalizations due to complications [10]. Finally, regarding long-term disease outcomes, the greatest concern with poor medication adherence lies in inducing tumor resistance and elevating the risk of recurrence and progression. When tumor cells are repeatedly exposed to drug concentrations insufficient to inhibit their growth, the development of tumor resistance becomes inevitable. This ultimately leads to treatment failure, increasing the risk of tumor recurrence and disease progression, thereby threatening the long-term survival of patients [11]. Consequently, effectively improving medication adherence among patients with cancer has become one of the most pressing challenges in contemporary oncology treatment management.

With the continuous advancement of mobile communication technology, mobile health (mHealth) has emerged as a novel health care service model that is being increasingly applied in chronic disease and cancer management. Utilizing wireless devices, such as smartphones, health monitoring wristbands, and smart pill bottles, mHealth provides services, including medication reminders, symptom monitoring, health education, and remote follow-ups, offering the advantages of cost-effectiveness, convenience, and unrestricted access regardless of time or location [12]. Recent studies indicate that mHealth interventions positively influence medication adherence among patients with cancer [13].

However, current research findings on the impact of mHealth interventions on medication adherence among patients with cancer remain inconsistent. Studies vary greatly in intervention formats and implementation durations, and their effectiveness across different cancer types remains unclear. Although a 2022 systematic review indicated that mHealth interventions can effectively improve medication adherence among chronic disease populations, including patients with cancer [14], systematic evaluations specifically targeting patients with cancer are currently lacking. Furthermore, this review did not perform subgroup analyses based on patient characteristics, intervention formats, or duration, leaving the impact of these variables on adherence improvement unclear. There is a critical need to systematically synthesize existing evidence to more accurately assess the practical efficacy of mHealth interventions in enhancing medication adherence among patients with cancer.

We aim to conduct a systematic review and meta-analysis to systematically integrate evidence on the impact of mHealth interventions on medication adherence among patients with cancer. Through subgroup analyses, we further explore the effects of intervention format, duration, and cancer type on intervention outcomes, thereby providing evidence-based guidance for clinical practice and mHealth intervention design.

## Methods

### Registration

This systematic review adheres to the PRISMA (Preferred Reporting Items for Systematic Reviews and Meta-Analyses) checklist (Checklist 1) and has been registered in PROSPERO (CRD42024522261) [15].

### Search Strategy

The initial literature search strategy for this systematic review was designed according to the preregistered protocol. However, the original protocol included only Chinese and English studies and used insufficient search terms, resulting in limitations of the search strategy. Therefore, we optimized the search strategy in the final draft to enhance comprehensiveness and reduce language bias. These modifications have been explicitly reported as post-hoc adjustments in accordance with research standards.

This systematic review’s literature search has been reported according to the PRISMA-S statement [16]. A combination of subject headings and free-text terms was used for systematic retrieval, covering the following databases and registries: PubMed (NCBI), Embase (Embase.com), Web of Science (Clarivate), Cochrane Library (Wiley), CINAHL (EBSCO), China National Knowledge Infrastructure (CNKI), CqVIP (VIP Information), Sinomed (China Biology Medicine Disc; CBMdisc Platform), and ClinicalTrials.gov (NLM). The literature search timeframe was set from database inception to June 28, 2025. To ensure the timeliness of the search results, the retrieval strategy was optimized and rerun on December 31, 2025, to incorporate newly published literature.

The search strategy for this systematic review was developed by refining and optimizing strategies from prior studies [17], and it underwent peer review by experts in library and information retrieval. Search parameters were set to include only randomized controlled trials (RCTs) and peer-reviewed articles, with no restrictions on publication year or language, and no predefined filters were applied. To maximize retrieval volume, snowball citation searching was performed. This involved screening the reference lists of the included studies (backward citation searching) as well as identifying and reviewing studies that cited the included articles (forward citation searching). It should be noted that the literature search for this review was conducted exclusively using the aforementioned method and did not include searches of printed conference proceedings or government websites. Detailed search strategies are provided in Multimedia Appendix 1.

### Inclusion and Exclusion Criteria

#### Inclusion Criteria

The inclusion criteria were predefined using the PICOS (participants, interventions, control/comparison measures, outcomes, study type) framework [15]. The criteria were as follows: (1) participants: patients with a pathologically confirmed cancer diagnosis who were currently taking medication; (2) interventions: mHealth interventions designed to improve medication adherence among patients with cancer, including apps, websites, and text messaging services; (3) control/comparison measures: placebo controls, routine or standard interventions, and other non-mHealth interventions; (4) outcomes: primary outcomes, including medication adherence rate and mean medication adherence score, and secondary outcomes, including health literacy, self-efficacy, symptom burden, and satisfaction; and (5) study type: studies with a RCT design.

#### Exclusion Criteria

We excluded the following studies: (1) non–peer-reviewed research, such as research proposals, conference abstracts, and reviews, typically lacking rigorous methodological validation; (2) those for which full-text access was unavailable; and (3) those lacking accessible or convertible medication adherence data.

### Definition and Classification of mHealth

The mHealth interventions included in this review varied in their implementation formats. To facilitate subsequent integration and comparison, we predefined the following classification criteria based on the primary delivery platform and user interaction model of the intervention content [18]:

1. Mobile apps: Interventions are primarily implemented through dedicated software installed on smartphones or tablets. Such apps can be accessed via mainstream app stores (eg, App Store and Google Play) or through links provided by the research team. They typically feature interactive interfaces (eg, menus, buttons, and data input fields) and support offline functionality for at least some features.
2. Websites: Interventions are primarily delivered through mobile device browsers or web applications (without requiring local software installation). Key characteristics include: access via URL, interfaces optimized for mobile screens (responsive design), and typical requirement of a persistent internet connection.
3. Text messaging services: Interventions are primarily delivered via short messaging or multimedia messaging systems, push text, or multimedia content. These services rely on cellular network messaging systems using asynchronous communication. Owing to character length limitations, the content is typically concise.

### Study Selection

After importing the retrieved studies into EndNote 20.6 (Clarivate Analytics) and removing duplicates using the software, 2 researchers (JHY and QYZ) were invited to cross-review the titles and abstracts to complete the initial screening and full-text reading of the remaining literature. When disagreements arose between the 2 researchers regarding inclusion decisions, a third researcher (WCL) was consulted to participate in discussions. Where necessary, the third researcher made the final determination on whether to include a study. For studies lacking medication adherence data or where full-text access was unavailable, we first attempted to contact the corresponding author to obtain the data. If the author did not respond or the data could not be obtained, the study was excluded from the meta-analysis.

### Data Extraction

For included studies, data were extracted using Microsoft Excel software, encompassing the following: (1) general information (eg, first author’s name, nationality, publication year, theoretical framework, study design, and background); (2) characteristics of patients with cancer (eg, sample size, mean age/age range, and disease type); (3) intervention characteristics (eg, intervention format, intervention content, intervention duration, and intervention frequency); and (4) primary outcomes and measurement tools.

### Definition and Classification of Outcome Indicators

#### Primary Outcome Indicators

Medication adherence was defined as the degree of alignment between a patient’s actual medication-taking behavior and the prescribed treatment regimen.

To clarify how this metric was measured across studies, we documented the following three operational dimensions during data extraction:

1. Measurement tools: Categorized into 1 of 5 types: electronic monitoring devices (eg, smart pillboxes), pharmacy dispensing/dispensing records, standardized self-assessment scales (eg, Morisky scale), researcher-designed questionnaires or interviews, and pill counting methods.
2. Data format and definition: Extracted each study’s specific criteria for defining “adherence” (eg, “number of missed doses in the past 7 days” or “proportion of days adhering to the prescription ≥80%”) and recorded the reporting format of results (continuous variable or dichotomous variable).
3. Processing procedures: For continuous data (eg, mean adherence score), directly extract mean, SD, and sample size; for dichotomous data (eg, adherence rate), extract the number of adherence events and the total sample size.

Based on this classification, we conducted separate meta-analyses for studies reporting continuous and dichotomous variables. This approach avoids introducing additional assumptions and potential bias from forced data format conversion. When pooling continuous data, since different adherence scoring tools (with varying scales) were used across studies, we selected the standardized mean difference (SMD) as the pooled effect measure. This allowed all studies to be included in a unified statistical comparison model. It is important to emphasize that while SMD primarily addresses statistical comparability, it does not eliminate potential conceptual differences underlying varying operational definitions. Such differences may influence the clinical interpretation of results, necessitating caution in analysis. For dichotomous variables, we selected the odds ratio (OR) as the effect measure owing to its greater prevalence and interpretability in epidemiological research.

#### Secondary Outcome Indicators

The secondary outcome indicators were as follows:

1. Symptom burden: The overall experience of patients with cancer regarding symptoms arising from the disease and treatment in terms of frequency, severity, and functional impact [19].
2. Self-efficacy: The strength of confidence patients with cancer have in their ability to successfully perform and adhere to specific disease management and treatment-related behaviors [20].
3. Service satisfaction: The level of satisfaction with health care services among patients with cancer [21].
4. Health literacy: The ability of patients with cancer to access, understand, evaluate, and apply health information to make appropriate health decisions for maintaining and promoting their own health [22].

### Risk of Bias, Level of Evidence, and Methodological Quality Assessment

Two researchers (QYZ and JHY) assessed the risk of bias in RCTs using the 7 domains of The Cochrane Collaboration’s tool for risk of bias assessment (version 2.0) [23]. The Cochrane risk of bias assessment tool encompasses 5 key domains: randomization process, intervention deviation, missing data, outcome assessment, and selective reporting. Risks within each domain are categorized as low risk, high risk, or unclear risk. Studies meeting low risk in all domains are classified as grade A, those meeting criteria in some domains are classified as grade B, and those failing to meet criteria in any domain are classified as grade C. This systematic review included only grade A or B literature, and evidence quality was assessed using GRADE Pro software (Evidence Prime Inc). This tool categorizes evidence quality into 4 levels: high, moderate, low, and very low. Factors leading to downgrading encompass 5 aspects: risk of bias, imprecision, inconsistency, indirectness, and publication bias. High-quality evidence indicates very high certainty that the true effect is close to the estimated effect; moderate-quality evidence reflects moderate confidence in the effect estimate, with the true effect potentially close to but possibly substantially different from the estimate; low-quality evidence indicates limited certainty about the effect estimate, with the true effect likely substantially different from the estimate; and very low–quality evidence indicates almost no confidence in the effect estimate, with the true effect highly likely to be substantially different from the estimate [24]. When 2 researchers (QYZ and JHY) disagreed on the risk of bias or evidence grade of a study, a third researcher (WCL) was consulted. If necessary, the third researcher made the final decision on whether to include the study.

### Data Analysis

A narrative synthesis approach was used to summarize study characteristics, including research design, publication year, country, participant characteristics, and intervention features. For outcome measures unsuitable for meta-analysis, a narrative synthesis approach was also applied for analysis.

Although the preregistration protocol specified the use of Stata (Version 17.0; StataCorp) and RevMan (Version 5.4; Cochrane) for statistical analysis, the final statistical analysis was conducted in RStudio (Posit) using a conservative random-effects model based on the assumption of between-study heterogeneity. Effect sizes were pooled using the Hartung-Knapp-Sidik-Jonkman (HKSJ) method [25]. To enhance methodological rigor and clinical interpretability, multiple analytical measures were introduced during the optimization phase: the Egger test was used to assess small-sample effects, 95% prediction intervals (PIs) were incorporated, τ^2^ was used to quantify heterogeneity, and the GRADE (Grading of Recommendations Assessment, Development, and Evaluation) approach was used to assess evidence quality [26].

For continuous variables, the SMD was used to calculate the pooled effect size, interpreted according to Cohen *d* criteria: 0.2, 0.5, and 0.8 represent small, moderate, and large effects, respectively. For dichotomous variables, the OR was used. Each pooled effect size is accompanied by a 95% CI. Additionally, we report PIs to quantify the variability of actual effects across different populations or settings [27].

The Cochrane Collaboration recommends the HKSJ method for random-effects meta-analysis [28]. When facing insufficient statistical power due to a small number of included studies, random-effects meta-analysis based on the HKSJ method outperforms the standard DerSimonian-Laird method [27]. By using a t-distribution and adjusting SEs using the q statistic, the HKSJ method provides more robust effect size CI estimates. Particularly with limited studies, HKSJ-estimated CIs are more accurate as they better reflect the inherent uncertainty and imprecision of effect estimates [29]. Model selection should be based on conceptual assumptions about the true effect distribution rather than descriptive statistics like *I*^2^ [26]. Given the presence of differences in participant characteristics, interventions, and measurement tools across the included studies, a conservative random-effects model was used for all analyses. This systematic review used restricted maximum likelihood estimation to account for between-study variance, thereby more accurately reflecting the impact of true heterogeneity on effect estimates. Heterogeneity was quantified using Q-tests, *I*^2^, and τ^2^ measures [27]. All statistical analyses were conducted in the R Studio environment using R software (version 4.5.2), utilizing packages including metafor, forestplot, and ggplot2.

Subgroup analyses of postintervention data were based on priority intervention criteria: (1) intervention duration, (2) cancer type, and (3) intervention type. Sensitivity analyses were conducted to assess result stability. Funnel plots were generated, and Egger regression intercept tests were applied to examine small-sample effects. Note that these methods aim to detect small-sample effects rather than directly assess publication bias [30,31]. Small-sample effects may stem from publication bias, selective reporting, genuine heterogeneity between studies, and methodological differences. According to methodological standards, funnel plots require at least 10 studies for reliable interpretation; otherwise, the reliability of interpretations is low [30]. Egger regression intercept analysis was applied to all outcome measures in this systematic review.

### Deviations From the Registered Protocol

This systematic review strictly followed the protocol registered in advance on the PROSPERO platform (CRD42024522261). During methodological refinement and peer review, we identified the following limitations in the original protocol and made two subsequent revisions accordingly:

1. Optimization of the search strategy: The original protocol initially proposed a search strategy combining subject headings with free-text terms. Following peer and expert review, we adopted recommendations to “expand search terms to reduce the risk of omission.” This involved supplementing search terms, such as adding “online*” and “drug therapy computer assisted,” to enhance search comprehensiveness.
2. Change in the statistical analysis tool: Although the original protocol specified Stata (version 17.0) and RevMan (version 5.4) for data analysis, during implementation, we ultimately selected RStudio to perform all statistical analyses. This decision was made to leverage RStudio’s flexibility in data visualization and statistical model implementation.

## Results

### Literature Search Results

**Figure 1.**
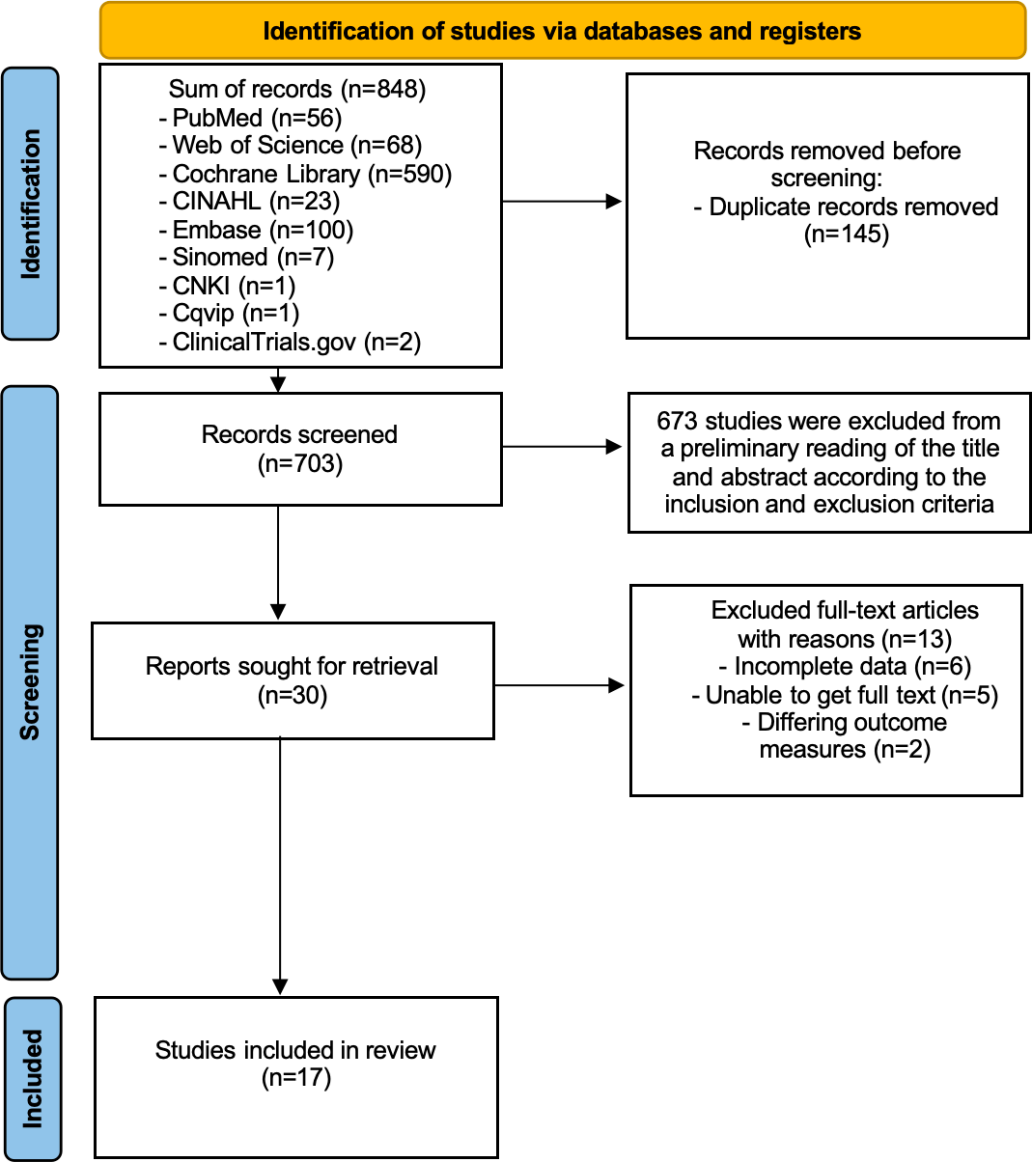
PRISMA (Preferred Reporting Items for Systematic Reviews and Meta-Analyses) flow diagram.

This literature search identified a total of 848 studies, including 846 from databases and clinical trial registries and 2 from reference lists. After deduplication, 703 studies were ultimately retained. Following preliminary screening of titles and abstracts against the inclusion criteria, 30 studies underwent full-text assessment. After full-text evaluation, 13 studies were excluded: 6 due to incomplete data, 5 due to unavailable full texts, and 2 due to differing outcome measures. Ultimately, 17 studies [19-22,32-44] were included (Figure 1).

### Characteristics of the Included Studies

This systematic review included 17 RCTs. Six studies were published in Simplified Chinese [20,21,36,37,42,44], while the remaining 11 were English-language publications [19,22,32-35,38-41,43], spanning publication dates from 2016 to 2025. Studies originated from 8 countries: the United States, China, Turkey, Australia, the Netherlands, South Korea, Iran, and Finland, and all were conducted in hospital and home settings.

The studies included a total of 1309 participants, with sample sizes ranging from 28 to 166 cases. Cancer types included breast cancer (3 studies) [33,39,43]; leukemia (4 studies) [22,35,40,41]; unspecified cancer types (7 studies) [19,32,34,37,38,42,44]; and thyroid cancer, pheochromocytoma, and prostate cancer (1 study each) [20,21,36].

Regarding intervention formats, the primary methods used were mobile apps (10 studies) [19,33,34,36,37,40-44], text messaging services (5 studies) [20,32,35,38,39], and websites (2 studies) [21,22]. Additionally, 2 studies utilized smart pill bottles as supplementary tools, integrated with either mobile apps or text messaging services [39,43]. Regarding intervention content, mobile apps and websites primarily enhanced medication adherence through online Q&A sessions, multiplayer online games, and science-popularization video pushes, while text messaging services focused on delivering personalized medication reminders.

Regarding intervention providers, 7 studies explicitly reported that physicians and/or nurses delivered interventions via mHealth platforms [20,21,37,39,40,42,44], while the remaining 10 did not specify the type of health care professional [19,22,32-36,38,41,43]. The intervention duration ranged from 3 weeks to 9 months, with only 3 studies reporting frequency (daily to 3 times weekly) [20,33,40]. Regarding theoretical frameworks, only 1 study designed interventions based on social cognitive theory [32].

All studies assigned control groups to receive usual care. Twelve studies described usual care components, including health education, medication guidance, and regular follow-up [19-21,32-40], while the remaining 5 did not provide specific details [22,41-44]. Detailed study characteristics are summarized in Table 1.

**Table 1.** Characteristics of the included studies.

Author (year), country	Study design	Theory	Study setting	Cancer type	Sample, n	Age (years)	Details of the experimental group (format, content, duration, and frequency)	Control group	Main outcomes	Outcome indicator
Spoelstra et al [32] (2016), United States	RCT^a^	Social cognitive theory	Home	Breast cancer and other cancers	69	Experimental: mean 60.1 (SD 10.1); Control: mean 59.9 (SD 11.2)	Format: text messaging service; Content: text message reminders to take patient cancer pills; Duration: 3 weeks; Frequency: NA^b^	Usual care	The difference in the score between the experimental and control groups was 0.29 for adherence, and the difference was not statistically significant.	Symptom burden: MDASI-C^c^; Medication adherence: pill count
Kim et al [33] (2018), Korea	RCT	—^d^	Hospital	Breast cancer	72	Experimental: 49.8; Control: 52.1	Format: app (mobile game); Content: education for preventing the side effects of anticancer drugs; Duration: 3 weeks; Frequency: 3 times a week, 30 minutes each time	Usual care	The mobile game group showed better drug adherence (MARS^e^; mean 7.6, SD 0.7 vs mean 6.5, SD 0.5; *P*<.001).	Medication adherence: MARS; Symptom burden: BREF^f^
Verweij et al [22] (2023), Netherlands	Cluster RCT	—	Hospital and home	Leukemia	86	Range: 18‐65	Format: website; Content: provides accurate and easy-to-understand information about the disease, medication, side effects, and the effect on daily life, and optimizes medication compliance; Duration: 6 months; Frequency: NA	Usual care	No statistical difference was found in the mean total score of patient medication compliance between groups (*P*=.922).	Health literacy: eHEALS^g^; Medication adherence: MARS; Symptom burden: EORTC QLQ-CML24^h^
Greer et al [19] (2020), United States	RCT	—	Hospital and home	Hematologic cancer, lung cancer, breast cancer, glioma, sarcoma, gastric cancer, genital cancers, and melanoma	166	Range: 21-88; Experimental: mean 52.85 (SD 13.74); Control: mean 53.76 (SD 12.08)	Format: app; Content: a personalized medication dosing schedule, an adherence and symptom reporting module, educational resources for symptom management and other cancer-related topics, and Fitbit integration for tracking physical activity; Duration: 12 weeks; Frequency: NA	Usual care	Patient-reported adherence (MMAS-4^i^), symptom burden, quality of life, satisfaction with treatment, and health care utilization did not differ significantly between groups.	Medication adherence: MMAS-4; Symptom burden: MDASI-C; Service satisfaction: FACIT-TS-PS^j^
Wang et al [21] (2020), China	RCT	—	Home	Abdominal aortic aneurysm	82	Range: 36-71; Experimental: mean 51.83 (SD 4.56); Control: mean 52.53 (SD 4.23)	Format: website/app; Content: health monitoring, entering, and querying; Duration: 6 months; Frequency: NA	Usual care	The medication compliance of the observation group was significantly higher than that of the control group (*P*<.05).	Medication adherence: self-made medication adherence questionnaire; Service satisfaction: self-made follow-up satisfaction survey questionnaire
Karaaslan-Eşer et al [34] (2021), Turkey	Cluster RCT	—	Hospital and home	Gastrointestinal cancers, lung cancer, breast cancer, and renal cancer	84	Experimental: mean 60.33 (SD 9.31); Control: mean 62.14 (SD 9.97)	Format: app; Content: providing information about oral anticancer agents and symptom-specific recommendations; Duration: 6 months; Frequency: NA	Usual care	The mean score of the intervention group increased over the first-, third-, and sixth-month measurements (*P*<.05).	Medication adherence: OCAS^k^; Symptom burden: MSAS^l^
Kekäl et al [35] (2016), Finland	RCT	—	Hospital	Leukemia	68	Range: 25‐83	Format: text messaging service; Content: patients were offered daily text messages to their mobile phones based on the dosing regimen of their treatment to help them remember the time to take their medication; Duration: 9 months; Frequency: NA	Usual care	The groups were similar in adherence as assessed by MMAS class (*P*>.05).	Medication adherence: MMAS
Wang et al [36] (2022), China	RCT	—	Hospital and home	Thyroid cancer	98	Experimental: mean 48.2 (SD 8.0); Control: mean 46.6 (SD 9.4)	Format: app; Content: send medication reminders; researchers monitor patients’ follow-up status in the background and provide timely reminders; Duration: 60 days; Frequency: NA	Usual care	The medication compliance of the study group was significantly better than that of the control group (*P*<.01).	Medication adherence: MMAS-8; Health literacy: Medication Belief Questionnaire
Che et al [37] (2022), China	RCT	—	Hospital and home	Pheochromocytoma	76	Range: 28-65; Experimental: mean 42.65 (SD 3.79); Control: mean 43.26 (SD 3.92)	Format: app; Content: includes a disease knowledge module, medical interaction module, cutting-edge information module, and appointment follow-up module; Duration: 4 weeks; Frequency: NA	Usual care	The medication adherence score was higher in the intervention group than in the control group (*P*<.05).	Medication adherence: MMAS-8
He et al [20] (2023), China	RCT	—	Hospital and home	Prostate cancer	80	Range: 61-87; Experimental: mean 74.95 (SD 6.78); Control: mean 72.05 (SD 6.56)	Format: text messaging service; Content: at 7:00 every morning, a greeting message and medication reminder are sent to the WeChat group; Duration: 6 months; Frequency: once a day	Usual care	At 1, 3, and 6 months, medication adherence was higher in the education group than in the control group, with statistically significant differences (*P*<.05).	Medication adherence: MMAS-8
Başoğlu et al [38] (2024), Turkey	RCT	—	Home	Colorectal cancer, breast cancer, and others	48	Experimental: mean 72.00 (SD 7.86); Control: mean 70.52 (SD 5.32)	Format: text messaging service; Content: send patients 1 educational video and 4 text messages per week via WhatsApp; Duration: 4 weeks; Frequency: NA	Usual care	There was no significant difference between the mean scores of the intervention and control groups regarding the pretest application of the OCAS (*P*>.05).	Medication adherence: OCAS
Graetz et al [39] (2024), United States	Cluster RCT	—	Hospital and home	Breast cancer	28	Range: 33-82; Experimental: mean 58.1 (SD 10.1); Control: mean 60.2 (SD 12.3)	Format: smart pill bottle + text messaging service; Content: text message reminders for missed or incorrect doses; smart pill bottle provides real-time medication adherence monitoring; Duration: 90 days; Frequency: NA	Usual care	Among participants who received the intervention, the average adherence rate was 79.1% for usual care participants and 89.0% for PRO^m^ participants (*P*=.05).	Medication adherence: Nomi smart pill bottle system; Symptom burden: self-made symptom burden questionnaire
Dang et al [40] (2025), Australia	RCT	—	Home	Leukemia	28	Range: 18‐65	Format: app; Content: medication-taking schedule, individualized daily medication reminders, and weekly side effect surveys; Duration: 12 weeks; Frequency: once a day	Usual care	There was no difference in the proportion of patients who had optimal adherence (medication rate of adherence ≥90%) between the intervention and control groups (100% for both groups).	Medication adherence: pharmacy refills; Symptom burden: FACT-G^n^; Self-efficacy: PRO
Hosseinpour et al [41] (2025), Iran	RCT	—	Hospital	Leukemia	70	Range: 10‐21	Format: app; Content: offers features such as updates (eg, adjusting the volume of alerts, selecting alert types, setting alarms based on dates, and adding games to encourage adolescent engagement); Duration: 8 weeks; Frequency: NA	Usual care	The adherence score in the intervention group differed significantly from that in the control group (*P*<.05).	Medication adherence: MARS
Lin et al [42] (2018), China	RCT	—	Home	Colorectal cancer, lung cancer, liver cancer, and others	97	Range: 29‐74	Format: app; Content: the app provides medication instructions, including information on usage methods, dosage, and side effects; Duration: 3 months; Frequency: NA	Usual care	Compared with the control group, the observation group demonstrated significantly higher compliance (*P*<.05).	Medication adherence: self-made medication adherence questionnaire; Self-efficacy: CPSS^o^
Park et al [43] (2022), Korea	RCT	—	Home	Breast cancer	57	Experimental: mean 52.07 (SD 9.34); Control: mean 54.74 (SD 7.87)	Format: app + smart pill bottle; Content: participants were reminded to take their medication as per the beeping and blinking function of the smart pill bottle cap, which indicated the scheduled time; they also received smartphone notifications that the time of taking the medication was recorded automatically; Duration: 4 weeks; Frequency: NA	Usual care	Mean medication adherence rates were higher in the experimental group than in the control group (*P*=.004).	Medication adherence: Pillsy mobile app; Self-efficacy: medication self-efficacy scale
Zhao et al [44] (2023), China	RCT	—	Home	NA	100	Experimental: mean 60.67 (SD 6.59); Control: mean 60.82 (SD 6.76)	Format: app; Content: remind patients to take their medication on time via mobile apps or text messages, and provide guidance on dosage, frequency, and other aspects to ensure patients take their medication accurately and on schedule; Duration: 3 months; Frequency: NA	Usual care	Following the intervention, the Morisky medication adherence scores of patients in the experimental group were higher than the scores of patients in the control group, with a statistically significant difference (*P*<.05).	Self-efficacy: SCS^p^; Medication adherence: MMAS-8

a RCT: randomized controlled trial.

b NA: not applicable.

c MDASI-C: M.D. Anderson Symptom Inventory.

d Not reported.

e MARS: Medication Adherence Rating Scale.

f BREF: The World Health Organization Quality of Life-BREF Scale.

g eHEALS: eHealth Literacy Scale.

h EORTC QLQ-CML24: European Organization for Research and Treatment of Cancer Quality of Life - Chronic Myeloid Leukemia 24-item.

i MMAS: Morisky Medication Adherence Scale.

j FACIT-TS-PS: Functional Assessment of Chronic Illness Treatment-Treatment Satisfaction-Patient Satisfaction.

k OCAS: Oral Chemotherapy Adherence Scale.

l MSAS: Memorial Symptom Assessment Scale.

m PRO: patient-reported outcomes.

n FACT-G: Functional Assessment of Cancer Therapy-General.

o CPSS: Chronic Pain Self-Efficacy Scale.

p SCS: Self-Control Scale.

### Risk of Bias in Studies

**Figure 2.**
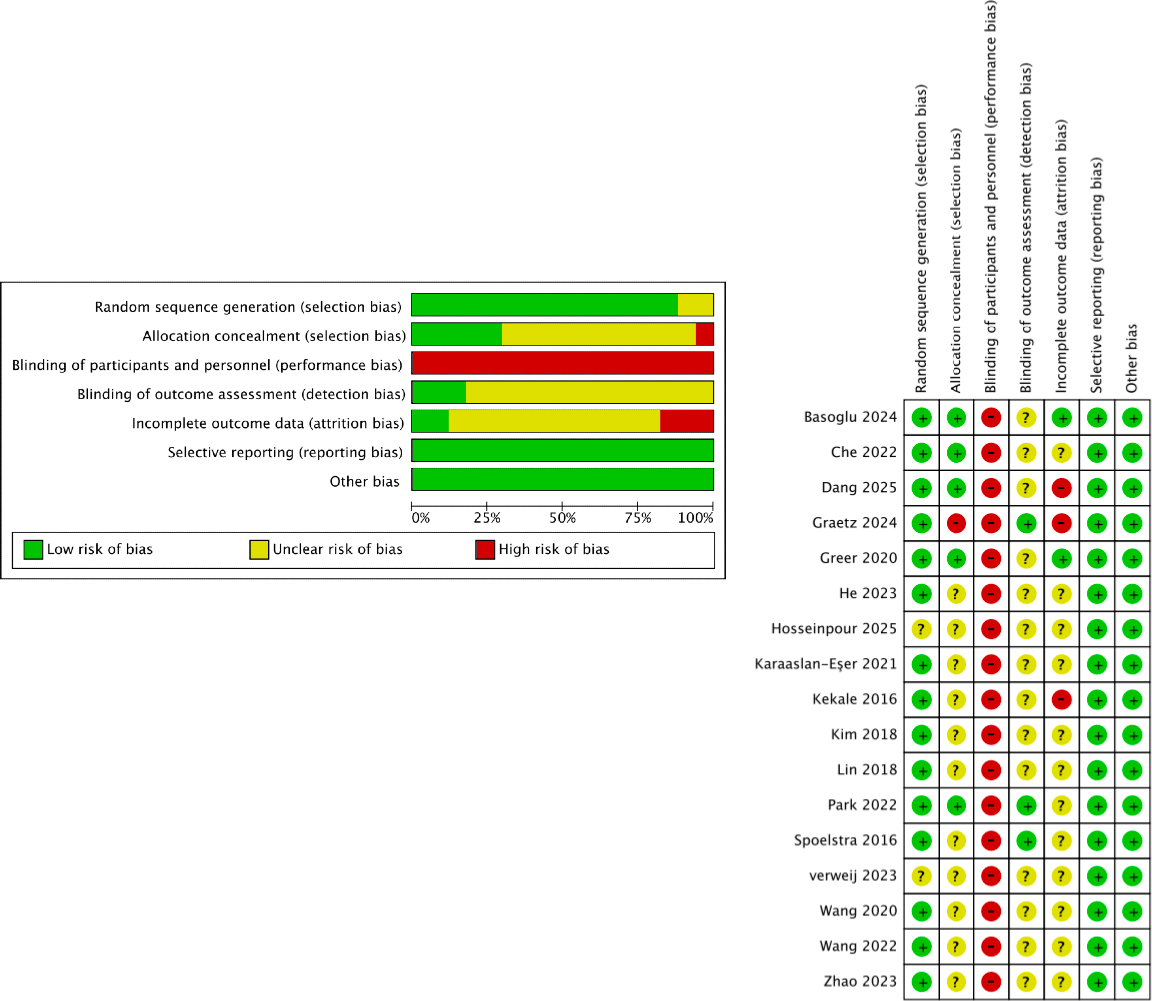
Risk of bias. (A) Risk of bias graph; (B) Risk of bias summary [19-22,32-44].

Ten studies reported randomization methods, while 5 described allocation concealment methods. Since improving medication adherence among patients with cancer using mHealth technology constitutes a behavioral intervention, blinding participants and researchers proved challenging. Consequently, none of the studies used blinding, resulting in a high risk of performance bias. Furthermore, as outcome measures were primarily assessed using subjective scales, the risk of measurement bias remains unclear. Only 2 studies used intention-to-treat analysis to address missing outcome data. No studies demonstrated selective reporting bias or other types of bias, resulting in an overall quality rating of grade B (Figure 2).

### Evidence Quality Assessment

This systematic review used the GRADE evidence rating tool to assess the quality of evidence for each outcome measure. Overall, evidence quality ranged from moderate to very low. For the impact of mHealth interventions on medication adherence rates, the evidence quality was moderate, downgraded primarily due to risk of bias and imprecise data. For the effect of mHealth on the mean medication adherence score, the evidence quality was very low, downgraded due to high risk of bias, inconsistent results, and imprecise data. For the effect of mHealth on symptom burden, the evidence quality was low, downgraded due to risk of bias and imprecise data.

Regarding the effect of mHealth on self-efficacy, the evidence quality was also low, downgraded due to risk of bias and imprecision. The GRADE evidence summaries for each outcome are presented in Table 2, with detailed downgrade justifications available in Multimedia Appendix 2.

**Table 2.** GRADE (Grading of Recommendations Assessment, Development, and Evaluation) evidence.

Outcomes	Studies (design), n	Risk of bias	Inconsistency	Indirectness	Imprecision	Publication bias	Patients^a^		Effect		Certainty
							Mobile health	Usual care	Relative, OR^b^ (95% CI)	Absolute, value (95% CI)	
Medication adherence rate	7 (RCT^c^)	Serious	Not serious	Not serious	Serious	Undetected	269/313 (85.9)	190/295 (64.4)	3.47 (1.92-6.26)	219 more per 1000 (from 132 more to 275 more)	Moderate
Mean medication adherence scores	10 (RCT)	Serious	Serious	Not serious	Serious	Undetected	361	340	—^d^	SMD^e^ 1.01 (0.51 to 1.52)	Very low
Symptom burden	7 (RCT)	Serious	Not serious	Not serious	Serious	Undetected	267	234	—	SMD −0.38 (−0.61 to −0.14)	Low
Self-efficacy	6 (RCT)	Serious	Not serious	Not serious	Serious	Undetected	214	191	—	SMD 0.98 (0.25 to 1.72)	Low

a Data are presented as n/N (%) or n.

b OR: odds ratio.

c RCT: randomized controlled trial.

d Not applicable.

e SMD: standardized mean difference.

### Meta-Analysis Results

#### Effect of mHealth on the Medication Adherence Rate

**Figure 3.**
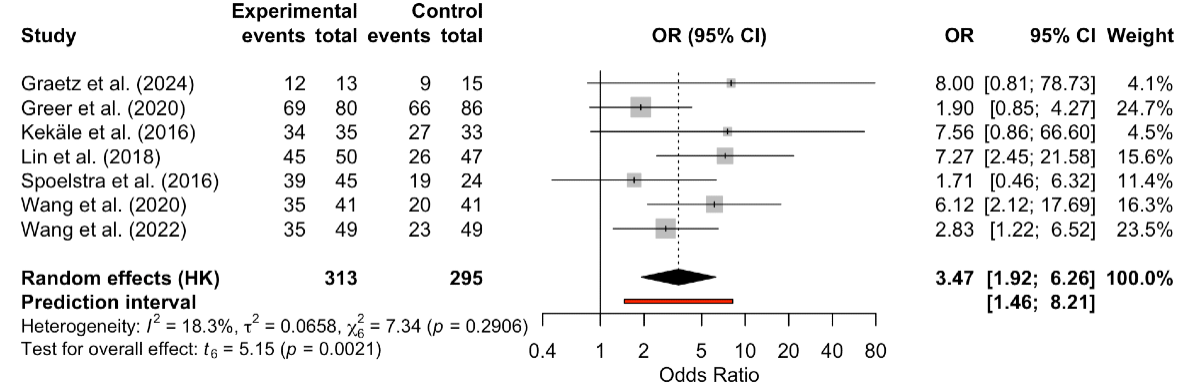
Forest plot of the effect of mobile health on the medication adherence rate [19,21,32,35,36,39,42]. OR: odds ratio.

Seven studies evaluated the impact of mHealth on the medication adherence rate [19,21,32,35,36,39,42]. Despite low heterogeneity (*I*^2^=18.3%; τ^2^=0.0658), we used a random-effects model to provide a more conservative and generalizable effect estimate. This decision was based on differences in adherence measures, intervention content, and measurement tools across the included studies. Pooled results demonstrated that mHealth significantly improved the medication adherence rate compared to controls (OR 3.47, 95% CI 1.92-6.26, 95% PI 1.46-8.21; *I*^2^=18.3%; τ^2^=0.0658; *P*=.002; Figure 3). The 95% CI confirmed the statistical significance of the average effect, with low heterogeneity among studies (*I*^2^=18.3%; τ^2^=0.0658). Furthermore, the 95% PI was entirely above 1, indicating that the benefit of mHealth on the medication adherence rate is likely reproducible in future studies. Note that the small number of included studies may render this 95% CI unreliable. The quality of evidence regarding mHealth’s impact on the medication adherence rate was moderate (Table 2 and Multimedia Appendix 2). Despite downgrading due to risk of bias and imprecision, mHealth still demonstrated a significant effect on the medication adherence rate.

#### Effect of mHealth on Mean Medication Adherence Scores

**Figure 4.**
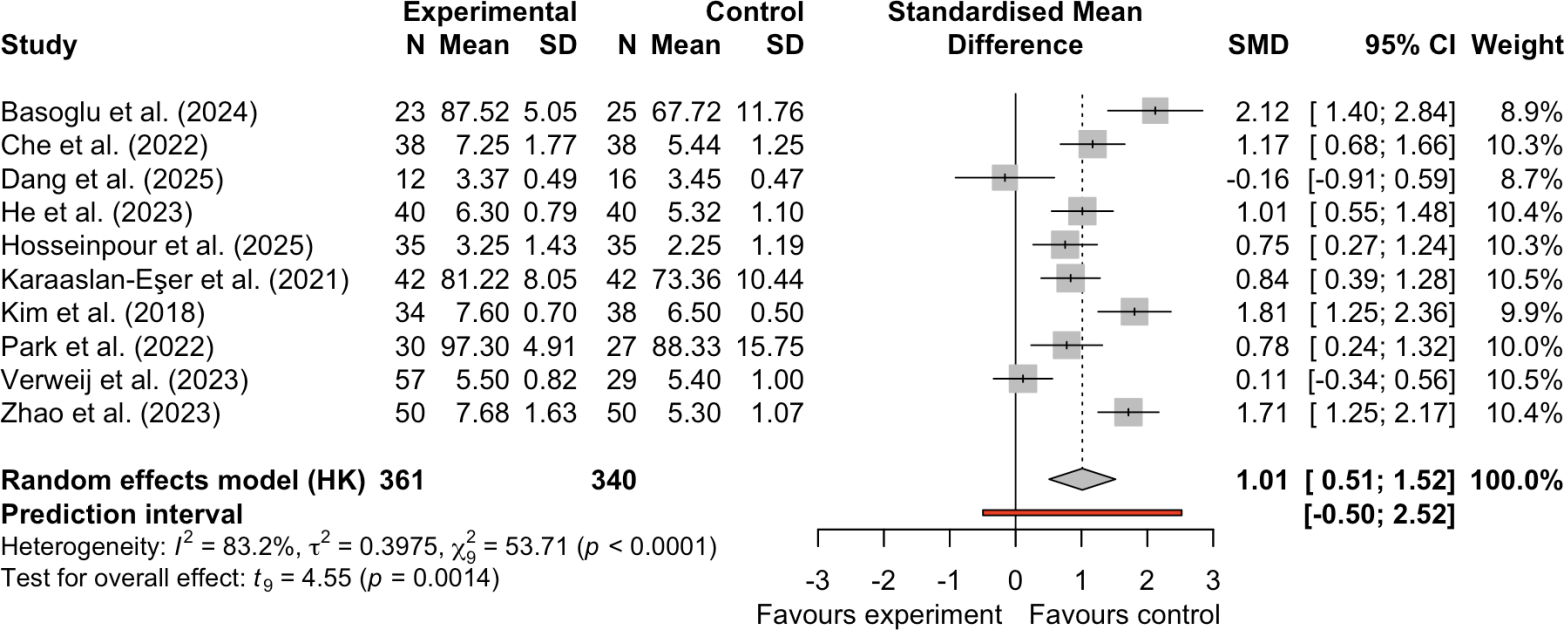
Forest plot of the effect of mobile health on mean medication adherence scores [20,22,33,34,37,38,40,41,43,44]. SMD: standardized mean difference.

Ten studies evaluated the impact of mHealth on mean medication adherence scores [20,22,33,34,37,38,40,41,43,44]. Given the high heterogeneity in the analyses (*I*^2^=83.2%; τ^2^=0.3975), we used a random-effects model for pooling to provide a more conservative and generally applicable effect estimate. The pooled analysis revealed that mHealth interventions significantly improved patients’ mean medication adherence scores (SMD 1.01, 95% CI 0.51-1.52; *I*^2^=83.2%; τ^2^=0.3975; *P*=.001; Figure 4). However, the PI (95% PI −0.50 to 2.52) for this effect estimate was wide and included zero, suggesting that the true effect may vary considerably around zero in future studies. This implies that mHealth may not yield significant effects for certain populations or in different contexts.

This result should be interpreted with caution. First, the extremely high heterogeneity (*I*^2^=83.2%; τ^2^=0.3975) indicates substantial differences in effect sizes across studies, potentially attributable to variations in intervention protocols, study populations, or measurement tools. Second, although the pooled effect was statistically significant, the quality of evidence for this outcome was rated as very low according to the GRADE criteria (Table 2 and Multimedia Appendix 2), primarily due to multiple downgrades for risk of bias, inconsistency, and imprecision. Therefore, while this analysis suggests that mHealth positively impacts average medication adherence scores, the certainty of this conclusion is constrained by the quality and heterogeneity of the existing evidence.

Sensitivity analyses by excluding individual studies showed that excluding the study by Dang et al [40] did not substantially alter the intervention effect on average medication adherence scores (SMD 1.12, 95% CI 0.64-1.60, 95% PI −0.24 to 2.49; *I*^2^=81.9%; τ^2^=0.3086; *P*<.001; see Figure S1 in Multimedia Appendix 3), further confirming the robustness of the original meta-analysis results.

To explore the effects of intervention duration, intervention type, and participant characteristics on mean medication adherence scores, we conducted exploratory subgroup analyses. Regarding intervention duration, the subgroup with duration <3 months (SMD 1.37, 95% CI 0.78-1.96; *P*=.001) showed a significantly greater effect than the subgroup with duration ≥3 months (SMD 0.49, 95% CI −0.39 to 1.37; *P*=.006), with a statistically significant between-group difference (*χ*²_1_=5.98; *P*=.01). Regarding intervention type, significant differences in effect size were observed among text messaging services (SMD 1.53, 95% CI −5.49 to 8.55; *P*=.01), apps (SMD 1.01, 95% CI 0.42-1.61; *P*<.001), and websites (SMD 0.11, 95% CI −0.34 to 0.56), and there were significant differences in effectiveness (*χ*²_2_=10.28, *P*=.006), with text messaging services yielding the highest point estimate but exhibiting an extremely wide CI. Subgroup differences by cancer type were also statistically significant (*χ*²_2_=8.86; *P*=.01). The breast cancer subgroup yielded the highest point estimate (SMD 1.29, 95% CI −5.25 to 7.83; *P*=.009), followed by other cancer types (SMD 1.09, 95% CI 0.10-2.08; *P*=.65) and leukemia (SMD 0.28, 95% CI −0.87 to 1.42; *P*=.07). It is important to emphasize that all analyses were post hoc exploratory comparisons, were not adjusted for randomization, and may be influenced by confounding factors (eg, shorter interventions potentially involving more frequent clinician-patient interactions). The observed intergroup differences represent only suggestive correlations and cannot be interpreted as causal relationships. Furthermore, limited numbers of studies and small sample sizes within some subgroups may have resulted in insufficient statistical power and unstable results. Therefore, these findings should be regarded as observational evidence for generating hypotheses and require further validation in future studies.

#### Effect of mHealth on Symptom Burden

**Figure 5.**
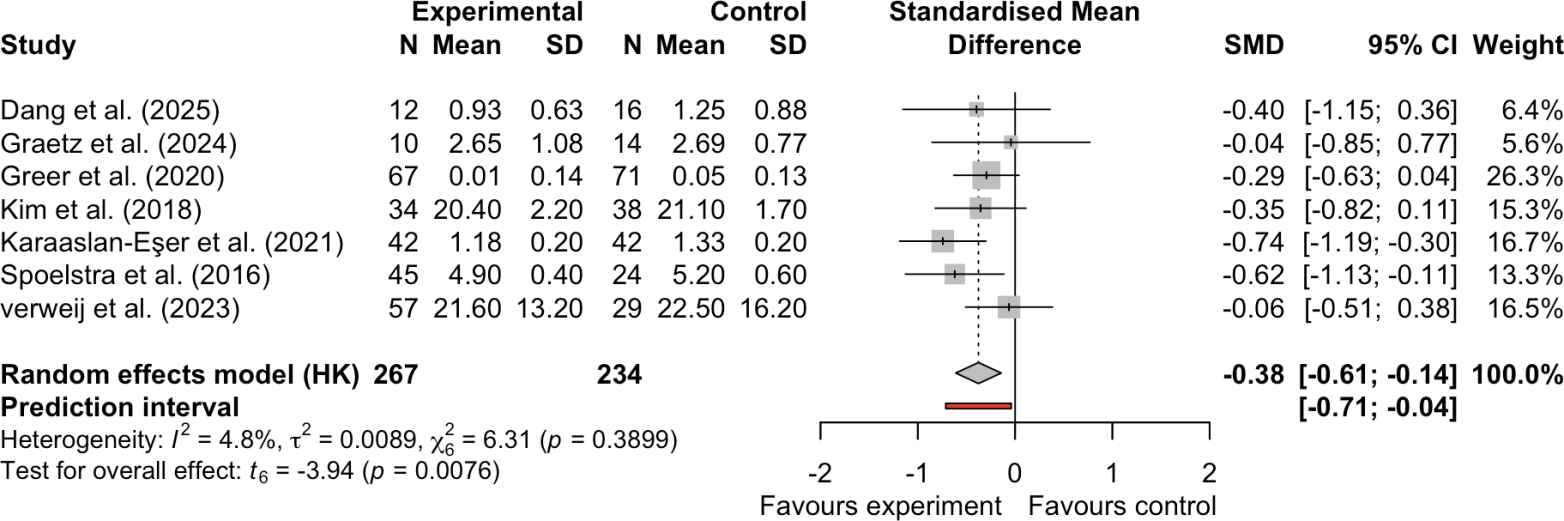
Forest plot of the effect of mobile health on symptom burden [19,22,32-34,39,40]. SMD: standardized mean difference.

Seven studies evaluated the impact of mHealth technologies on symptom burden in patients with cancer [19,22,32-34,39,40]. Despite low heterogeneity (*I*^2^=4.8%; τ^2^=0.0089), we used a random-effects model to obtain a more conservative and generalizable effect estimate. This decision was based on the variability in intervention content and measurement tools across the included studies. Compared with controls, mHealth interventions yielded modest effects on symptom burden improvement (SMD −0.38, 95% CI −0.61 to −0.14, 95% PI −0.71 to −0.04; *I*^2^=4.8%; τ^2^=0.0089; *P*=.008; Figure 5). The 95% CI confirmed the statistical significance of the pooled effect, with low study heterogeneity (*I*^2^=4.8%). Furthermore, the 95% PI did not cross zero, indicating that the effect of mHealth on symptom burden improvement is likely reproducible in future studies. Note that the small number of included studies may render this 95% PI unreliable. The quality of evidence regarding mHealth’s impact on symptom burden was rated as low (Table 2 and Multimedia Appendix 2). This downgrade resulted from risk of bias and imprecision, indicating a small effect of mHealth on symptom burden.

#### Effect of mHealth on Self-Efficacy

**Figure 6.**
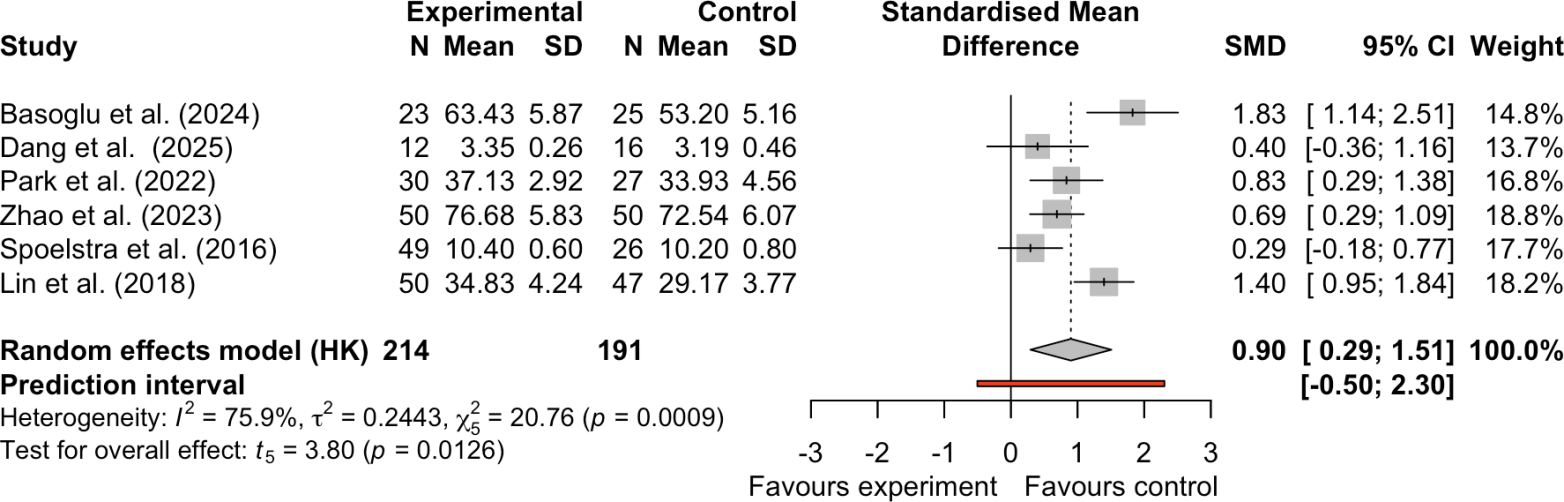
Forest plot of the effect of mobile health on self-efficacy [32,38,40,42-44]. SMD: standardized mean difference.

Six studies evaluated the impact of mHealth on self-efficacy among patients with cancer [32,38,40,42-44]. Due to high heterogeneity in this meta-analysis (*I*^2^=75.9%; τ^2^=0.2443), we used a random-effects model to obtain a more conservative and generalizable effect estimate. Pooled results demonstrated that mHealth significantly enhanced patient self-efficacy compared with controls (SMD 0.90, 95% CI 0.29-1.51, 95% PI −0.50 to 2.30; *I*^2^=75.9%; τ^2^=0.2443; *P*=.01; Figure 6). The 95% CI confirmed the statistical significance of the average effect, but the 95% PI spanned zero, indicating that mHealth may not improve self-efficacy in future studies or different populations. Note that the small number of included studies may render this 95% PI unreliable. The evidence quality for self-efficacy was low (Table 2 and Multimedia Appendix 2). The evidence rating was downgraded due to risk of bias and imprecision. mHealth still significantly influenced self-efficacy. Sensitivity analysis by excluding individual studies showed that removing the study by Dang et al [40] did not substantially alter the effect of mHealth interventions on mean self-efficacy scores (SMD 0.98, 95% CI 0.25-1.72, 95% PI −0.63 to 2.59; *I*^2^=81.9%; τ^2^=0.268; *P*=.02; Figure S5 in Multimedia Appendix 3), further confirming the robust stability of the original meta-analysis results (Figure 6).

#### Effect of mHealth on Service Satisfaction

Two studies evaluated the impact of mHealth on service satisfaction, but neither provided the mean and SD data required for a meta-analysis of service satisfaction. After multiple attempts to contact the corresponding authors of these studies, the requested data could not be obtained. Therefore, we conducted a descriptive analysis of the available data [19,21].

Wang et al [21] reported that during the 2-month follow-up period after the intervention, patients were surveyed using a satisfaction questionnaire developed by the hospital’s mHealth platform. The results showed significantly higher satisfaction scores in the intervention group compared with the control group. In contrast, Greer et al [19] used the validated Functional Assessment of Chronic Illness Treatment-Therapy Satisfaction-Patient Satisfaction (FACIT-TS-PS) scale to assess patient satisfaction, finding no statistically significant difference between intervention and control group scores.

#### Effect of mHealth on Health Literacy

**Figure 7.**
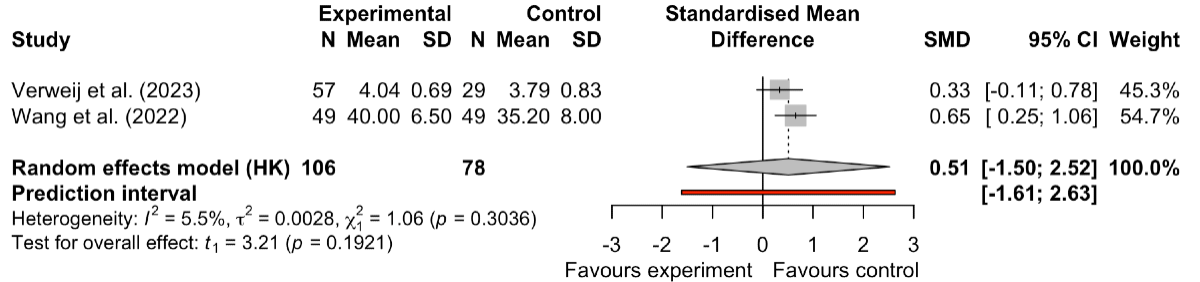
Forest plot of the effect of mobile health on health literacy [22,36]. SMD: standardized mean difference.

Two studies assessed health literacy levels [22,36]. Despite low heterogeneity (*I*^2^=5.5%; τ^2^=0.0028), given the differing measurement tools used across studies (Verweij et al [22] assessed patient health literacy using the validated eHealth Literacy Scale; Wang et al [36] used the validated Medication Beliefs Questionnaire), we used a random-effects model to obtain a more conservative and generalizable effect estimate. The meta-analysis revealed no statistically significant effect of interventions on health literacy (SMD 0.51, 95% CI −1.50 to 2.52; *I*^2^=5.5%; τ^2^=0.0028; *P*=.29; Figure 7). However, this result must be interpreted with caution for the following primary reasons. First, the inclusion of only 2 studies severely limited the statistical power of the analysis and the reliability of its conclusions. Second, the PI (95% PI −1.61 to 2.63) and heterogeneity parameter (τ^2^=0.0028) calculated from this limited number of studies were highly unstable, rendering them incapable of providing meaningful estimates for future study effects or true heterogeneity.

### Small Study Effects Assessment

To assess the small-sample effect, we followed the methodological guidance by Sterne et al [30], combining visual inspection of funnel plots with the Egger regression intercept test to analyze 4 outcome measures: mean medication adherence scores, medication adherence, symptom burden, and self-efficacy.

It should be noted that, except for the mean medication adherence score, fewer than 10 studies were included for each of the other outcome measures, which is below the ideal threshold for reliable assessment proposed by Lau et al [45]. Nevertheless, funnel plots were constructed for each measure (Figures S6-S9 in Multimedia Appendix 3) for preliminary exploration. Visual inspection revealed no obvious asymmetry in the funnel plots; however, this visual assessment itself carries considerable uncertainty due to the limited number of studies.

Subsequent quantitative analysis using the Egger test also failed to indicate statistically significant small-sample effects (medication adherence average score: z=1.64; *P*=.75; symptom burden: z=0.18; *P*=.91; self-efficacy: z=1.29; *P*=.78; and medication adherence rate: z=1.53; *P*=.26).

However, these findings must be interpreted with caution. The primary limitation lies in the insufficient number of included studies. First, a small sample size significantly reduces the statistical power of the Egger test, increasing the risk of type II error (ie, failing to detect a genuinely existing small-sample effect) [30]. Second, neither the symmetry of the funnel plot nor the nonsignificance of the Egger test provides sufficient evidence to rule out publication bias [45]. Furthermore, funnel plot asymmetry itself may stem from clinical heterogeneity or differences in methodological quality rather than specifically indicating publication bias. Therefore, based on the current limited data, the findings of this systematic review cannot rule out the possibility of potential publication bias or other forms of selective reporting bias.

## Discussion

### Summary of Research Findings

This systematic review aimed to systematically evaluate the impact of mHealth interventions on medication adherence among patients with cancer. The results indicate that mHealth interventions can improve medication adherence rates and mean adherence scores among patients with cancer. They also demonstrate positive effects in reducing symptom burden, enhancing self-efficacy, and increasing service satisfaction. However, mHealth showed no significant impact on health literacy. Subgroup analysis results indicate that the effectiveness of mHealth interventions is influenced by intervention timing, intervention type, and cancer type. Furthermore, variations in the intervention protocols and assessment tools used across the included studies may have impacted the reliability of the findings.

### mHealth Effectively Improves Medication Adherence

Regarding the medication adherence rate, the 95% PI following the mHealth intervention ranged from 1.46 to 8.21, indicating that the benefits of mHealth for the medication adherence rate are reproducible in future studies. In terms of the mean medication adherence score, the mHealth intervention demonstrated a significant effect, consistent with previous research findings [46]. From the perspective of specific effects and real-world effect variability, the 95% PI for the mean medication adherence score ranged from −0.50 to 2.52, encompassing zero. This indicates that while this study demonstrates a positive impact of mHealth interventions on the mean medication adherence score, the effect may not be significant in certain populations or different contexts.

Furthermore, the included studies utilized specific channels, such as mobile apps, websites, and text messaging services, to deliver knowledge and skills related to oral medication through diverse formats, including real-time online Q&A sessions, multiplayer online games, educational videos, and push notifications. These interventions also provided timely medication reminders. Both the content and methods effectively enhanced medication adherence among patients with cancer by addressing intrinsic knowledge and skill acquisition as well as extrinsic behavioral supervision. Based on this, it can be inferred that improved medication adherence contributes to optimizing the overall effectiveness of cancer treatment, such as reducing symptom burden and enhancing patient self-efficacy. Data analysis results also support this inference, showing that mHealth interventions not only significantly reduced patients’ symptom burden but also improved their self-efficacy and service satisfaction. However, the impact of mHealth interventions on health literacy was not statistically significant (*P*=.29), contradicting previous research findings [47]. This discrepancy may stem from the inclusion of only 2 studies in the health literacy outcome analysis, severely limiting statistical power and reducing the reliability of conclusions. Finally, this systematic review used subgroup analyses to examine the effects of intervention format, timing, and cancer type on mHealth intervention outcomes.

### Text Messaging Services Have the Best Intervention Outcomes

Mobile apps and websites offer health education information in diverse formats, including videos, audio, and text. Moreover, the information exchange between these platforms and patients with cancer—that is, between information providers and recipients—is often bidirectional and interactive. In contrast, text messaging services, which are somewhat outdated, provide information in a relatively monotonous format dominated by text, and the information transmission through this channel is predominantly one-way [48]. Theoretically, mobile apps and websites should yield superior intervention outcomes compared with text messages. However, our subgroup analysis revealed that among the 3 mHealth intervention formats, text messaging services demonstrated greater effectiveness than mobile apps and websites. The findings of this systematic review align with the research by Boima et al [49], which demonstrated that text messaging interventions were more effective than mobile apps and web-based interventions in reducing medication nonadherence events among patients with cardiovascular diseases.

The underlying reason for this discrepancy may be related to the age characteristics of patients with cancer. As shown in Table 1, the average age of patients included in this systematic review was relatively high, with 6 studies having an average age exceeding 55 years [20,32,34,38,39,44] and 2 studies having an average age exceeding 70 years [20,38]. Although text messaging services represent a simple, one-way communication method, their content is often concise, clear, and straightforward, making it easier to understand and implement. This characteristic reduces cognitive load during information reception and processing, potentially aligning better with the information format preferences and cognitive functional characteristics of older patients with cancer. Furthermore, compared with mobile apps and websites, text messaging services impose lower demands on mobile devices and network connectivity, enhancing their practical feasibility and applicability. These factors may explain why text messaging interventions outperformed mobile app and website interventions among patients with cancer, who are predominantly older adults.

This finding also raises the following questions: Have existing mHealth interventions delivered via mobile apps and websites sufficiently considered the cognitive characteristics and information preferences of older adults during their design and development? Could this oversight inadvertently contribute to and exacerbate the so-called digital divide?

### The Optimal Intervention Duration for mHealth is Less Than 3 Months

This systematic review conducted subgroup analyses based on different intervention durations using 3 months as the cutoff point. The selection of this cutoff point was primarily based on 2 considerations. First, from the perspective of clinical treatment patterns, the duration of oral therapy for patients with cancer typically ranges from 12 to 24 weeks. Three months corresponds to the midpoint of the treatment course, marking a critical transition from the early to the later stages of therapy [50]. Second, prior studies indicate that medication adherence often declines around 3 months after initiation due to factors like drug side effects and forgetfulness [8]. Subgroup analysis revealed that mHealth interventions lasting <3 months significantly improved the mean medication adherence scores of patients with cancer compared with those lasting ≥3 months. The findings of this systematic review align with the study by Al-Arkee et al [51], which demonstrated that mHealth interventions lasting <3 months were more effective than those lasting ≥3 months in improving the average medication adherence scores of patients with cardiovascular diseases.

From a behavioral science perspective, the superior effectiveness of mHealth interventions lasting <3 months, compared with those lasting ≥3 months, may stem from the behavioral habituation cycle. Research by Lally et al [52] indicates that the median time required to establish a new behavior is 66 days. This suggests that the critical phase for behavioral acquisition occurs mainly within the first 2 to 3 months. During this window, mHealth interventions can effectively promote the formation and consolidation of medication-taking behaviors. They do so by providing timely reminders, feedback, and external frameworks. Once the target behavior is automated, continued external prompts may offer diminishing marginal benefits [53]. After habit formation, long-term interventions (≥3 months) typically offer few additional benefits. In some cases, they may even reduce effectiveness by reinforcing external dependence or causing intervention fatigue. In summary, mHealth interventions of <3 months match the natural process of habit formation. By maximizing gains during the most sensitive adaptation period, they significantly improve medication adherence.

### mHealth Interventions Show the Most Effective Outcomes in Improving Medication Adherence Among Patients With Breast Cancer

This systematic review included subgroup analyses for different cancer types. Notably, the results indicate that mHealth interventions demonstrate greater efficacy in improving medication adherence among patients with breast cancer compared to patients with leukemia and those with other cancer types. However, no studies have specifically examined the impact of cancer type on the effectiveness of mHealth interventions in enhancing medication adherence. Therefore, future research should conduct in-depth analyses of the differential effects of such interventions across various cancer types.

### Limitations

This systematic review has several limitations. First, it included only 17 studies, and the small sample size may lead to imprecise 95% CIs and biased estimates, thereby weakening the reliability of the results. Additionally, the limited number of studies resulted in insufficient statistical power, potentially overlooking potential small-sample effects. Although the existing evidence is insufficient to prove the existence of small-sample effects, we cannot completely rule out the influence of publication bias or selective reporting bias.

Second, among the 17 included studies, only the study by Spoelstra et al [32] used a theoretical model (social cognitive theory) to guide the design of the mHealth platform. Interventions targeting medication adherence are fundamentally interventions targeting medication-taking behavior. From a behavioral science perspective, it is necessary to leverage well-established health behavior theoretical models to explore and analyze the underlying logic and influencing factors of medication adherence among patients with cancer. This approach aims to uncover the core drivers behind medication-taking behavior, thereby enabling the development of more precise and effective medication adherence intervention strategies.

Furthermore, all 17 included studies used uniform interventions for all patients. No tailored interventions were designed or implemented based on individual patient characteristics. Future design of mHealth strategies for cancer medication adherence should incorporate methods, such as latent profile analysis, user profiling, and in-depth interviews, to thoroughly analyze patient traits and needs. Integrating patient profiles into platform design will enable personalized, precision-based scientific interventions [54,55].

### Conclusions

Building upon previous systematic reviews, this systematic review systematically reviewed and comprehensively analyzed the impact of mHealth interventions on medication adherence among patients with cancer. Overall, existing evidence suggests that mHealth interventions hold potential advantages in improving medication adherence, self-efficacy, and service satisfaction, as well as reducing symptom burden. However, considerable heterogeneity exists across certain outcome measures, and the overall evidence quality remains moderate to low. Additionally, the included studies exhibited a moderate risk of bias, necessitating cautious interpretation of these findings. It is currently inconclusive to assert superiority over conventional care.

Despite these limitations, the significance of this systematic review lies not only in confirming the efficacy of mHealth interventions for improving medication adherence among patients with cancer but also in revealing their strategic value within the context of digital health care transformation. As chronic disease management shifts from hospital-centered to patient-centered models, mHealth care offers a viable pathway for achieving continuous care, personalized support, and low-cost, scalable interventions. Future research should enhance methodological rigor by conducting multicenter, large-sample, high-quality RCTs. These studies should explore the long-term efficacy and cost-effectiveness of mHealth care across diverse health care settings and patient populations to clarify its role and value within comprehensive cancer management systems.

## Supplementary material

10.2196/85949 Multimedia Appendix 1 Search strategy.

10.2196/85949 Multimedia Appendix 2 GRADE (Grading of Recommendations Assessment, Development, and Evaluation) evidence quality assessment.

10.2196/85949 Multimedia Appendix 3 Subgroup analysis, sensitivity analysis plots, and funnel plots for all results.

10.2196/85949 Checklist 1 PRISMA checklist.

## Abbreviations:

GRADE: Grading of Recommendations Assessment, Development, and Evaluation
HKSJ: Hartung-Knapp-Sidik-Jonkman
mHealth: mobile health
OR: odds ratio
PI: prediction interval
PRISMA: Preferred Reporting Items for Systematic Reviews and Meta-Analyses
RCT: randomized controlled trial
SMD: standardized mean difference
