# Supplementary material for: Effectiveness of Mobile Health for Improving Medication Adherence in Patients With Cancer: Systematic Review and Meta-Analysis of Randomized Controlled Trials
Source: J Med Internet Res. 2026 Mar 30;28:e85949. doi: 10.2196/85949 (PMC13035083; doi:10.2196/85949)
Supplement: Multimedia Appendix 1 [file jmir-v28-e85949-s001.docx]

Multimedia Appendix 1. Search strategy.

| Database | Search strategy | Results |
| --- | --- | --- |
| Pubmed (NCBI) | ("Telemedicine"[MeSH Terms] OR ("mHealth"[Title/Abstract] OR "mobile health"[Title/Abstract] OR "Telehealth"[Title/Abstract] OR "eHealth"[Title/Abstract] OR "mobile application*"[Title/Abstract] OR "website"[Title/Abstract] OR "online*"[Title/Abstract] OR "drug therapy computer assisted"[Title/Abstract])) AND ("Neoplasms"[MeSH Terms] OR ("tumo*"[Title/Abstract] OR "neoplas*"[Title/Abstract] OR "cancer*"[Title/Abstract] OR "malignanc*"[Title/Abstract] OR "malignant neoplasm*"[Title/Abstract])) AND ("Medication Adherence"[MeSH Terms] OR ("drug adherence"[Title/Abstract] OR "prescription adherence*"[Title/Abstract] OR "medication persistence"[Title/Abstract] OR "medication compliance"[Title/Abstract] OR "drug compliance"[Title/Abstract])) AND ("randomized controlled trial"[Publication Type] OR "randomized controlled trial"[Title/Abstract]) | 56 |
| Web of Science | ((TS=(Telemedicine)) OR AB=(mHealth OR mobile health OR Telehealth OR eHealth OR mobile application* OR website OR online* OR drug therapy computer assisted)) OR TI=(mHealth OR mobile health OR Telehealth OR eHealth OR mobile application* OR website OR online* OR drug therapy computer assisted) AND ((TS=(Neoplasms)) OR AB=(tumo* OR neoplas* OR cancer* OR malignanc* OR malignant neoplasm*)) OR TI=(tumo* OR neoplas* OR cancer* OR malignanc* OR malignant neoplasm*) AND ((TS=(Medication Adherence)) OR AB=(drug adherence OR prescription adherence* OR medication persistence OR medication compliance OR drug compliance)) OR TI=(drug adherence OR prescription adherence* OR medication persistence OR medication compliance OR drug compliance) AND TS=(Randomized controlled trial) | 68 |
| Cochrane Library | #1: MeSH descriptor: [Neoplasms] explode all trees  #2: tumo* OR neoplas* OR cancer* OR malignanc* OR malignant neoplasm*  #3: #1 OR #2  #4:(Medication Adherence OR drug adherence OR prescription adherence* OR medication persistence OR medication compliance OR drug compliance):ti,ab,kw (Word variations have been searched)  #5:(Telemedicine OR mHealth OR mobile health OR Telehealth OR eHealth OR mobile application* OR website OR online* OR drug therapy computer assisted):ti,ab,kw (Word variations have been searched)  #6:(Randomized controlled trial):ti,ab,kw (Word variations have been searched)  #7: #3 AND #4 AND #5 AND #6 | 590 |
| CINAHL | (Telemedicine or mHealth or mobile health or Telehealth or eHealth or mobile application* or website or online* or drug therapy computer assisted) AND (tumo* OR neoplas* OR cancer* OR malignanc* OR malignant neoplasm*) AND (Medication Adherence or drug adherence or prescription adherence* or medication persistence or medication compliance or drug compliance) AND (randomized controlled trials or rtc or randomised control trials or clinical controlled trial or clinical trial) | 23 |
| Embase | #1: 'medication compliance'/exp  #2: 'medication adherence':ti,ab,kw OR 'drug adherence':ti,ab,kw OR 'prescription adherence':ti,ab,kw OR 'medication persistence':ti,ab,kw OR 'drug compliance':ti,ab,kw  #3: #1 OR #2  #4: 'telemedicine'/exp  #5: 'mhealth':ti,ab,kw OR 'mobile health':ti,ab,kw OR 'telehealth':ti,ab,kw OR 'ehealth':ti,ab,kw OR 'mobile application*':ti,ab,kw OR 'website':ti,ab,kw OR 'online*':ti,ab,kw OR 'drug therapy computer assisted':ti,ab,kw  #6: #4 OR #5  #7: 'neoplasm'/exp  #8: 'tumo*':ab,ti,kw OR 'neoplas*':ab,ti,kw OR 'cancer*':ab,ti,kw OR 'malignanc*':ab,ti,kw OR 'malignant neoplasm*':ab,ti,kw  #9: #7 OR #8  #10: 'randomized controlled trial'/de  #11: #3 AND #6 AND #9 AND #10 | 100 |
| Sinomed | (("药物依从性"[常用字段:智能] OR "用药依从性"[常用字段:智能]) OR ("服药依从性"[不加权:扩展])) AND (("癌症"[常用字段:智能] OR "癌"[常用字段:智能] OR "恶性肿瘤"[常用字段:智能] OR "肿瘤"[常用字段:智能]) OR ("肿瘤"[不加权:扩展])) AND (("移动医疗"[常用字段:智能] OR "电子医疗"[常用字段:智能] OR "移动健康"[常用字段:智能] OR "电子健康"[常用字段:智能] OR "网站"[常用字段:智能] OR "应用程序"[常用字段:智能]) OR ("远程医学"[不加权:扩展])) | 7 |
| CNKI | SU=(移动健康 + 电子健康 + 移动医疗 + 电子医疗 + 网站 + 应用程序 + 远程医学) AND SU=(服药依从性 + 用药依从性 + 药物依从性) AND SU=(癌症 + 癌 + 肿瘤 + 恶性肿瘤) | 1 |
| Cqvip | M=(移动健康 OR 电子健康 OR 移动医疗 OR 电子医疗 OR 网站 OR 应用程序 OR 远程医学) AND M=(服药依从性 OR 用药依从性 OR 药物依从性) AND M=(癌症 OR 癌 OR 肿瘤 OR 恶性肿瘤) | 1 |
| ClinicalTrials.gov | (Telemedicine or mHealth or mobile health or Telehealth or eHealth or mobile application* or website or online* or drug therapy computer assisted) AND (tumo* OR neoplas* OR cancer* OR malignanc* OR malignant neoplasm*) AND (Medication Adherence or drug adherence or prescription adherence* or medication persistence or medication compliance or drug compliance) AND (randomized controlled trials or rtc or randomised control trials or clinical controlled trial or clinical trial) | 2 |

Note: The last search dates for the above databases were all December 31, 2025.
