# Supplementary material for: Effectiveness of Mobile Health for Improving Medication Adherence in Patients With Cancer: Systematic Review and Meta-Analysis of Randomized Controlled Trials
Source: J Med Internet Res. 2026 Mar 30;28:e85949. doi: 10.2196/85949 (PMC13035083; doi:10.2196/85949)
Supplement: Multimedia Appendix 2 [file jmir-v28-e85949-s002.docx]

**Author(s): Weichao Liu, Liping Wang, Qianyu Zhang, Jinghan Yang**

**Question:** Mobile health compared to usual care for medication adherence among cancer patients

**Setting: hosptial & home**

**Bibliography:**

| **Certainty assessment** | | | | | | | **№ of patients** | | **Effect** | | **Certainty** | **Importance** |
| --- | --- | --- | --- | --- | --- | --- | --- | --- | --- | --- | --- | --- |
| **№ of studies** | **Study design** | **Risk of bias** | **Inconsistency** | **Indirectness** | **Imprecision** | **Other considerations** | **mobile health** | **usual care** | **Relative (95% CI)** | **Absolute (95% CI)** |  |  |
| **medication adherence rate** | | | | | | | | | | | | |
| 7 | randomised trials | serious^a,b,c^ | not serious | not serious | serious^d^ | strong association | 269/313 (85.9%) | 190/295 (64.4%) | **OR 3.47** (1.92 to 6.26) | **219 more per 1,000** (from 132 more to 275 more) | ⨁⨁⨁◯ Moderate^a,b,c,d^ | CRITICAL |
| **mean medication adherence scores** | | | | | | | | | | | | |
| 10 | randomised trials | serious^a,b,c^ | serious^e^ | not serious | serious^d^ | none | 361 | 340 | - | SMD **1 SD higher** (0.5 higher to 1.5 higher) | ⨁◯◯◯ Very low^a,b,c,d,e^ | CRITICAL |
| **symptom burden** | | | | | | | | | | | | |
| 7 | randomised trials | serious^a,b,c^ | not serious | not serious | serious^d^ | none | 267 | 234 | - | SMD **0.35 SD lower** (0.6 lower to 0.1 lower) | ⨁⨁◯◯ Low^a,b,c,d^ | CRITICAL |
| **Self efficacy** | | | | | | | | | | | | |
| 6 | randomised trials | serious^a,b,c^ | not serious | not serious | serious^d^ | none | 214 | 191 | - | SMD **0.85 SD higher** (0.25 higher to 1.5 higher) | ⨁⨁◯◯ Low^a,b,c,d^ | CRITICAL |

**CI:** confidence interval; **OR:** odds ratio; **SMD:** standardised mean difference

#### Explanations

a. a Since using mobile health to improve medication adherence among cancer patients constitutes a behavioral intervention, blinding participants and researchers is challenging; none of the studies conducted blinding, posing a high risk of performance bias.

b. b Outcome indicators are measured through subjective scales, electronic monitoring, or pharmacy refills, and there is also uncertainty regarding the risk of bias in outcome measurement.

c. c Most studies did not adequately describe allocation concealment and outcome assessor blinding.

d. d In terms of sample size, there are significant differences among the studies. Some studies have relatively small sample sizes, which may lead to wider confidence intervals for effect estimates, making the estimates less precise.

e. e Differences in research design, such as intervention measures, study populations, and outcome indicators, have led to one study showing significant inconsistencies in results compared to the others.
