# Supplementary material for: Effectiveness of Mobile Health for Improving Medication Adherence in Patients With Cancer: Systematic Review and Meta-Analysis of Randomized Controlled Trials
Source: J Med Internet Res. 2026 Mar 30;28:e85949. doi: 10.2196/85949 (PMC13035083; doi:10.2196/85949)
Supplement: Multimedia Appendix 3 [file jmir-v28-e85949-s003.docx]

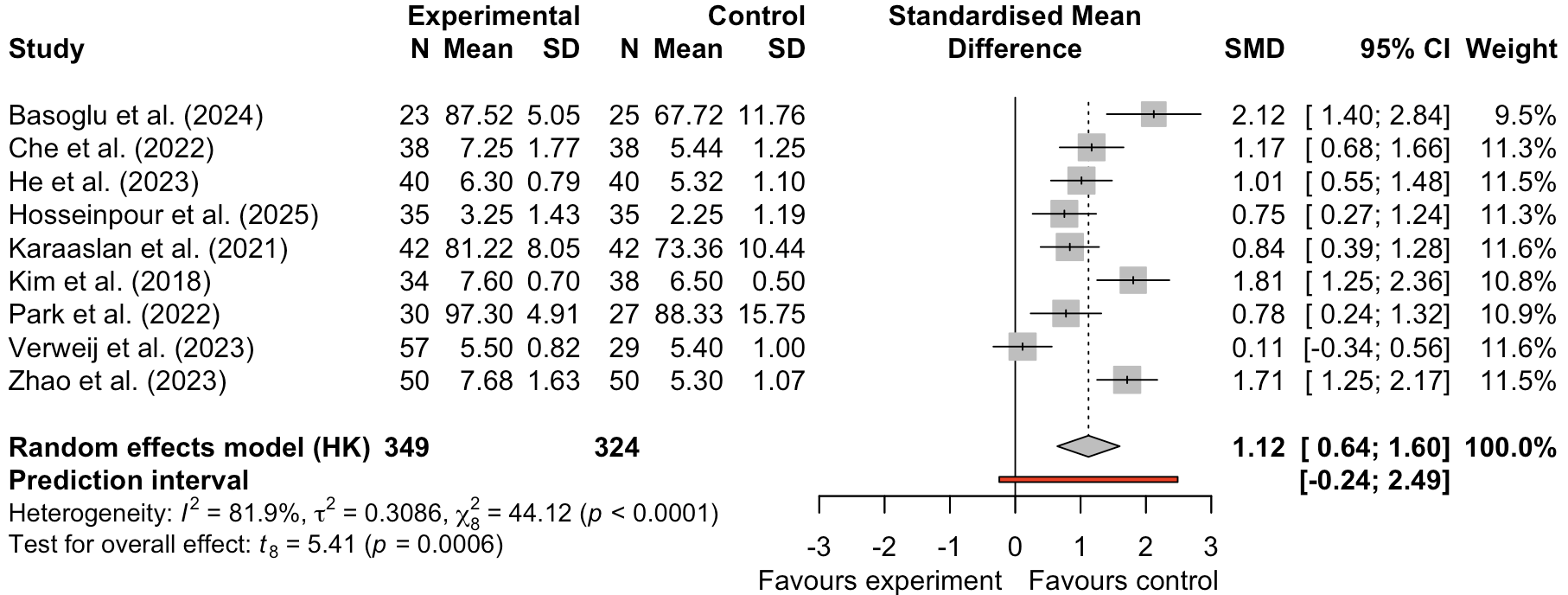


**Figure S1.** Forest plot of mean medication adherence scores after excluding a single study.


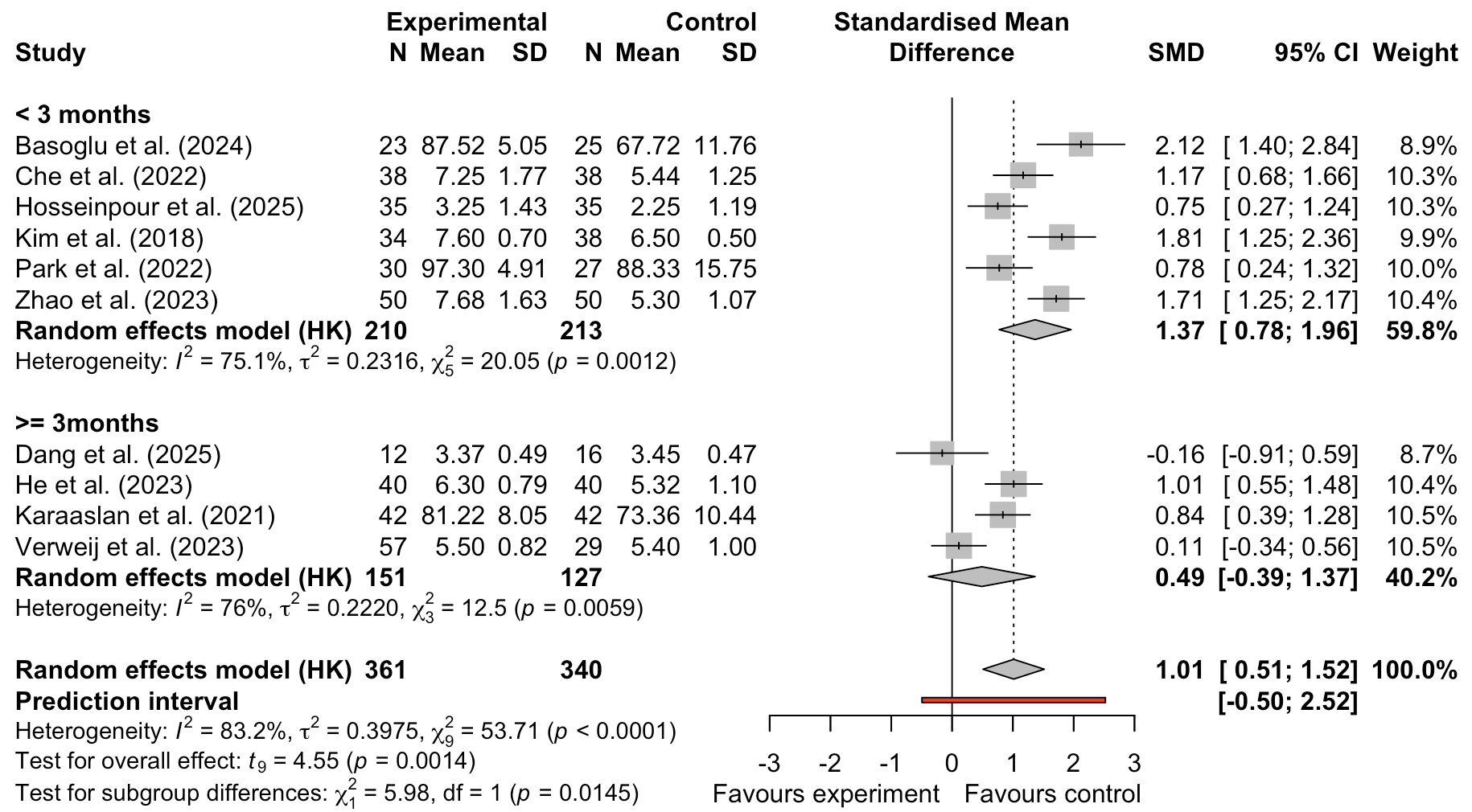


**Figure S2.** Forest plot of mean medication adherence scores in the intervention duration subgroup analysis.


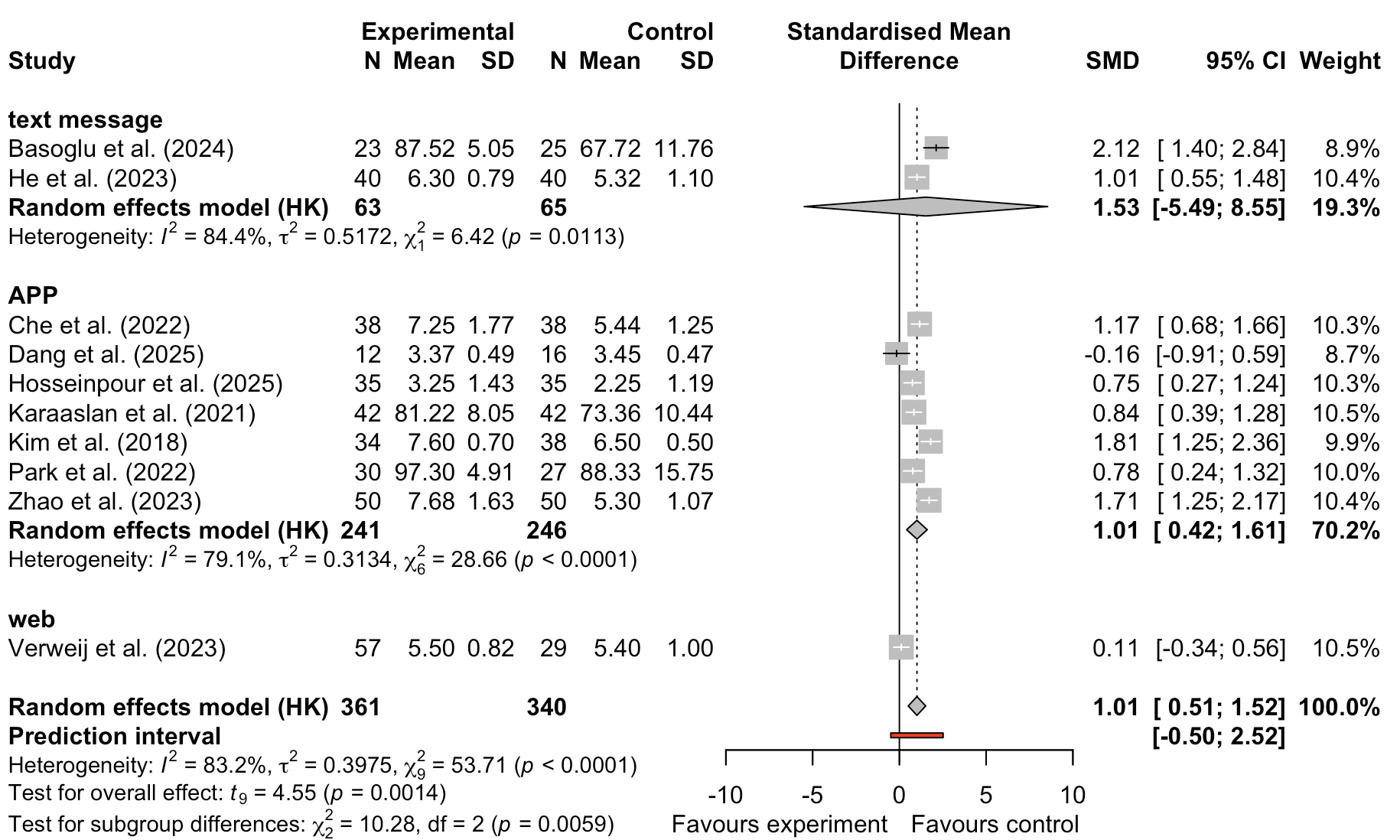


**Figure S3.** Forest plot of mean medication adherence scores in the intervention type subgroup analysis.


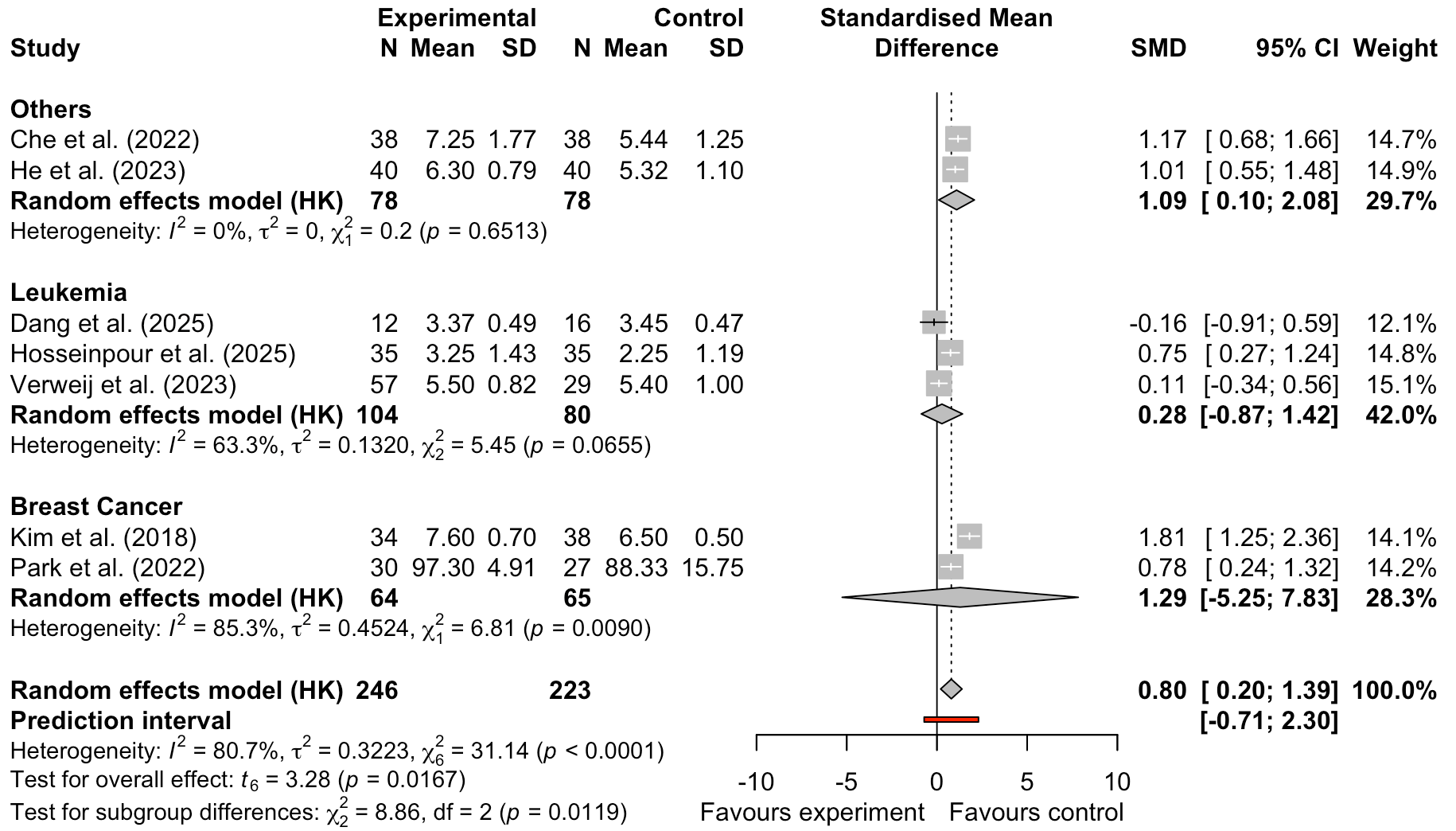


**Figure S4.** Forest plot of mean medication adherence scores in the cancer type subgroup analysis.


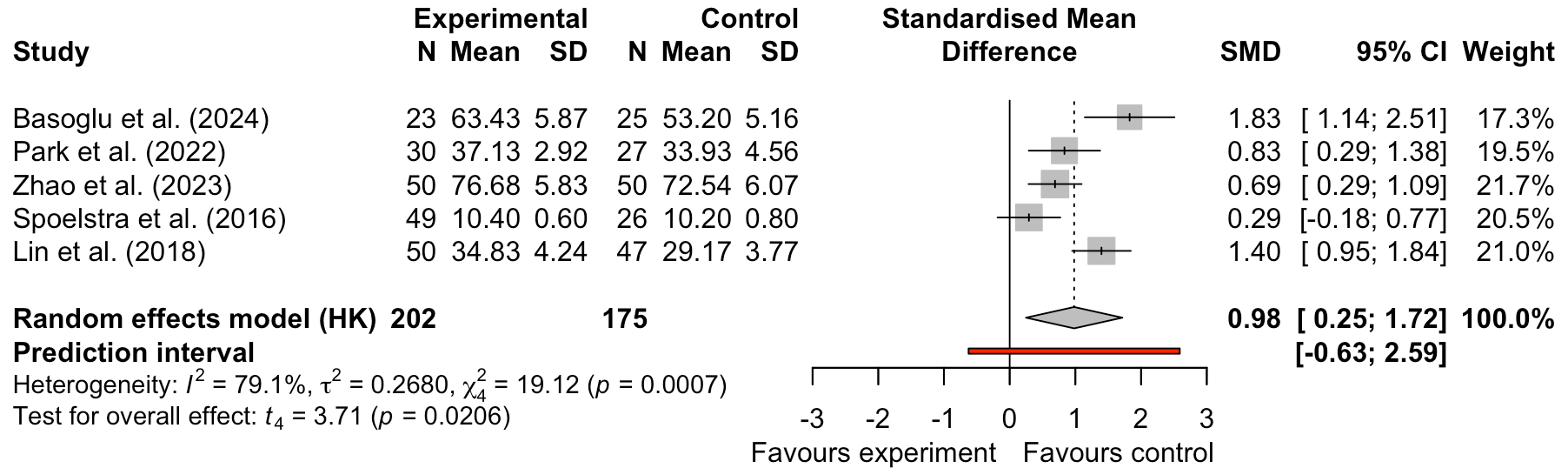


**Figure S5.** Forest plot of Self efficacy after excluding a single study.


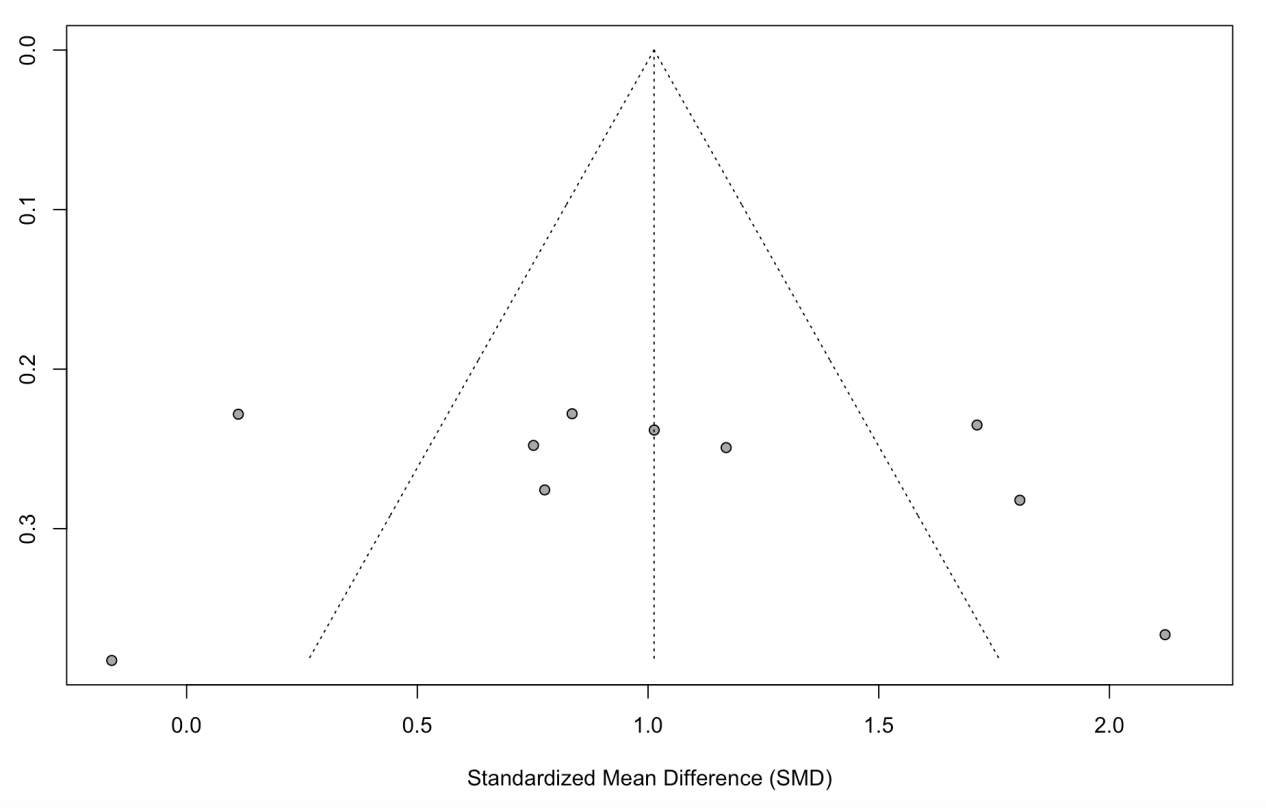


**Figure S6.** Funnel plot of mean medication adherence scores.


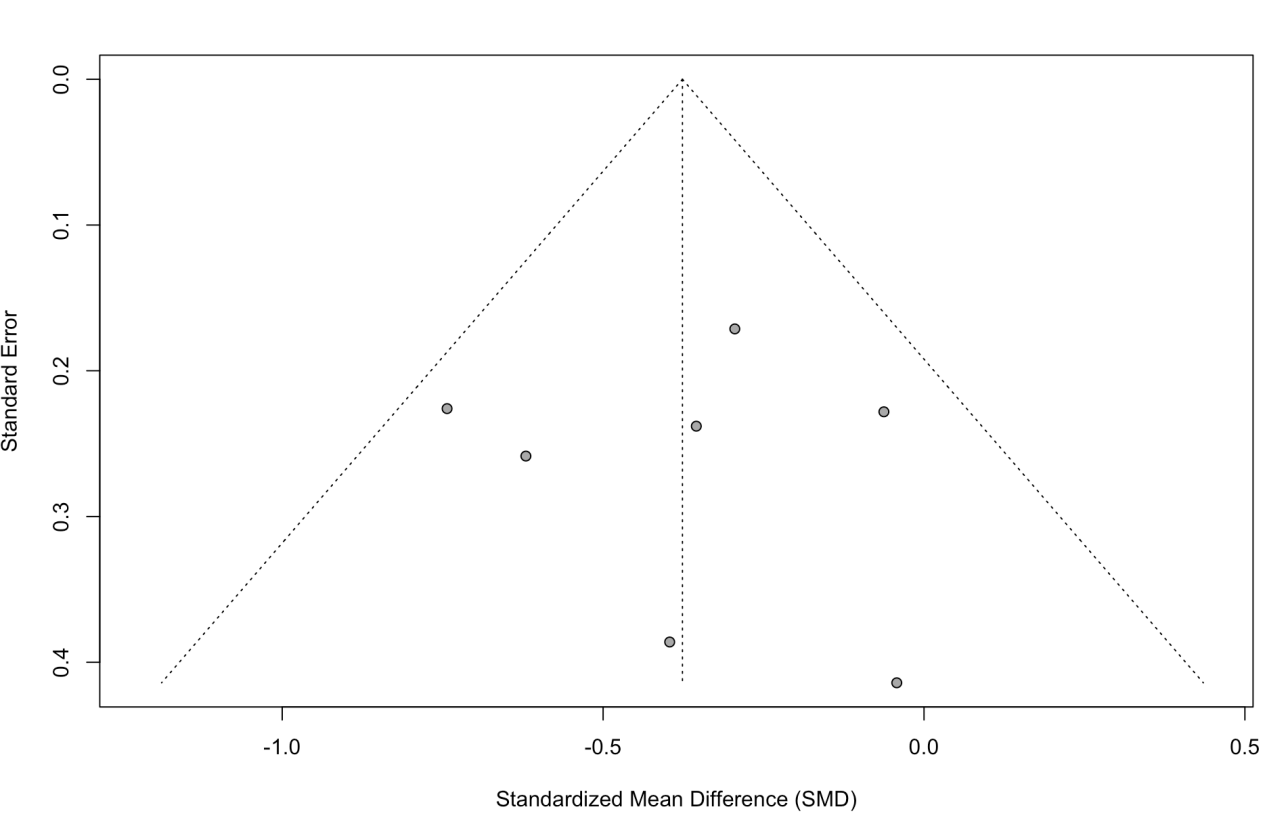


**Figure S7.** Funnel plot of symptom burden.


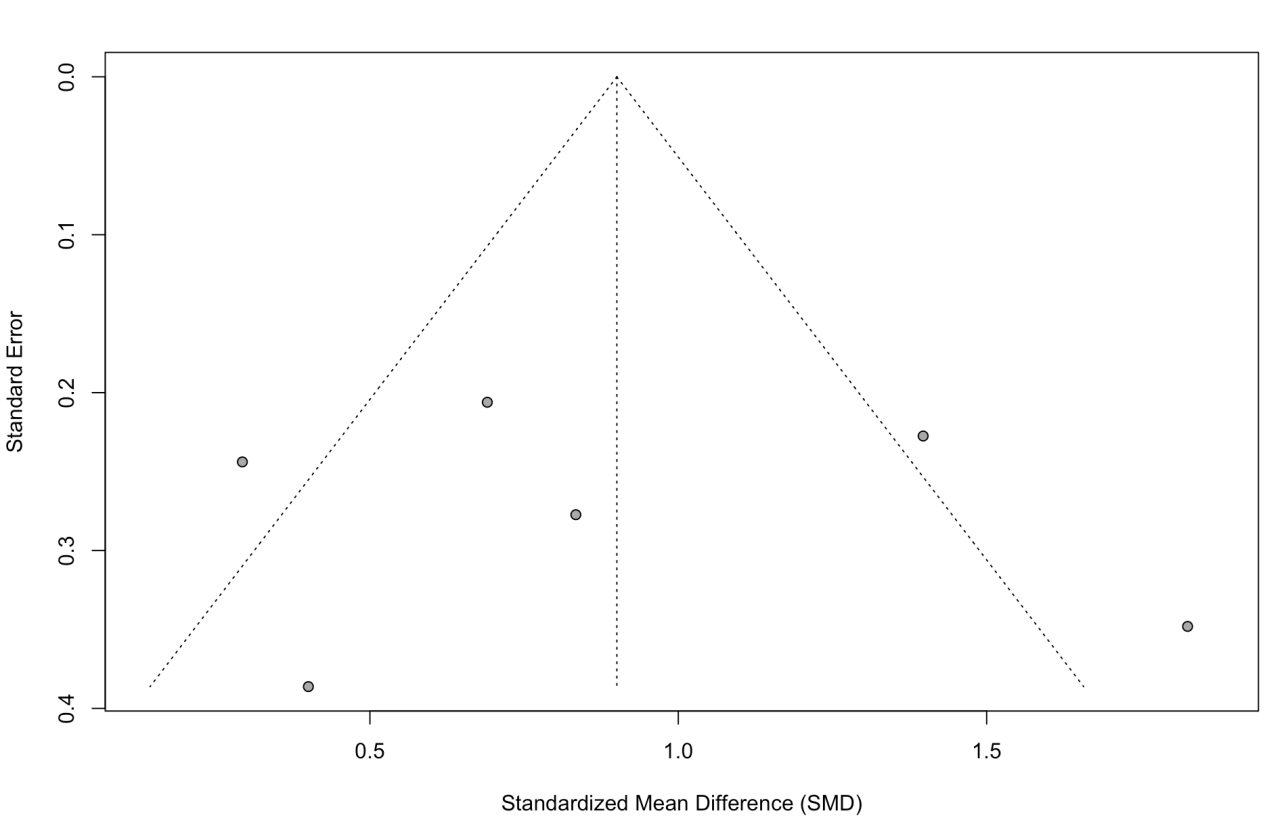


**Figure S8.** Funnel plot of self efficacy.


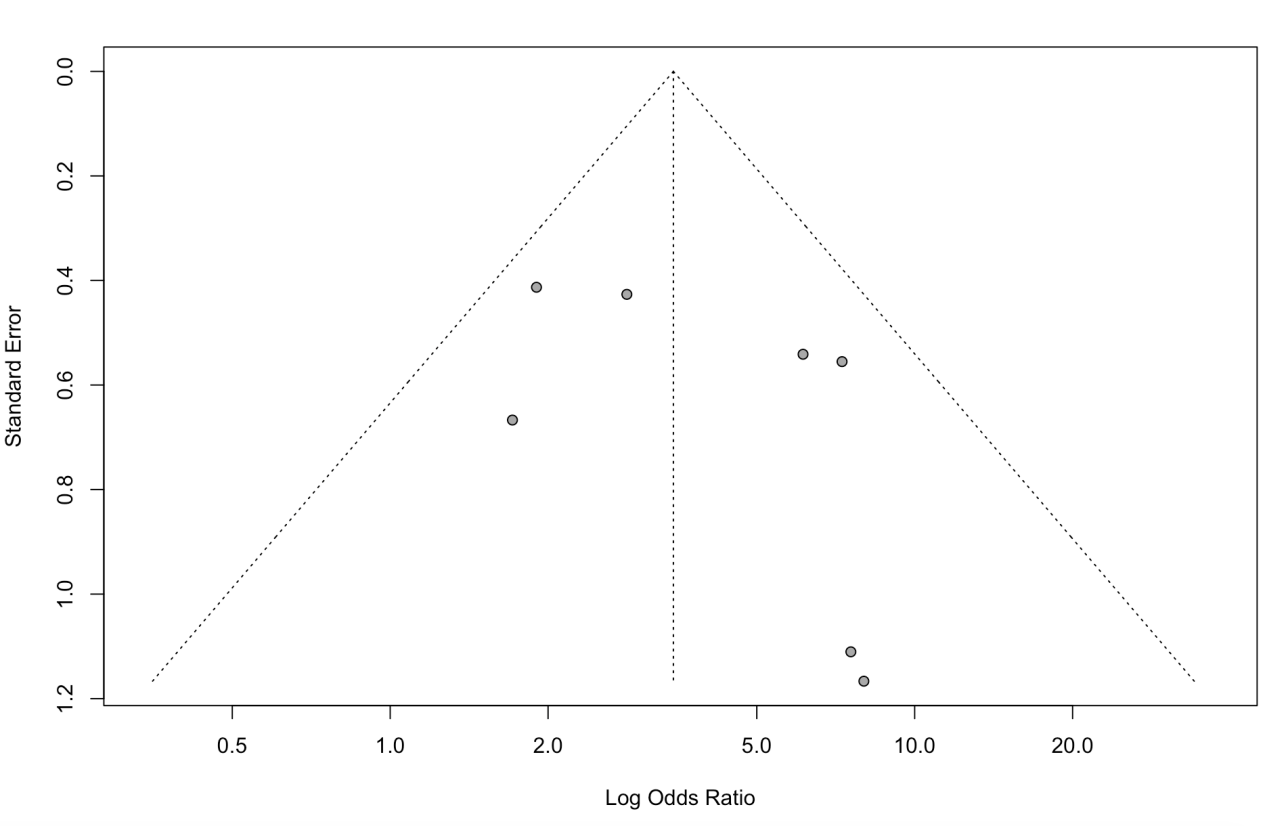
**Figure S9.** Funnel plot of medication adherence rate.
